# Genome-wide association study of disease resilience traits from a natural polymicrobial disease challenge model in pigs identifies the importance of the major histocompatibility complex region

**DOI:** 10.1093/g3journal/jkab441

**Published:** 2021-12-28

**Authors:** Jian Cheng, Rohan Fernando, Hao Cheng, Stephen D Kachman, KyuSang Lim, John C S Harding, Michael K Dyck, Frederic Fortin, Graham S Plastow, PigGen Canada, Jack C M Dekkers

**Affiliations:** 1 Department of Animal Science, Iowa State University, Ames, IA 50011, USA; 2 Department of Animal Science, University of California, Davis, Davis, CA 95616, USA; 3 Department of Statistics, University of Nebraska-Lincoln, Lincoln, NE 68583, USA; 4 Department of Large Animal Clinical Sciences, University of Saskatchewan, Saskatoon, SK S7N 5B4, Canada; 5 Department of Agricultural, Food and Nutritional Science, University of Alberta, Edmonton, AB T6G 2R3, Canada; 6 Centre de Développement du Porc du Québec Inc., Québec City, QC G1V 4M6, Canada; 7 PigGen Canada Research Consortium, Guelph, ON N1H4G8, Canada

**Keywords:** pig, disease resilience, genome-wide association studies, major histocompatibility complex

## Abstract

Infectious diseases cause tremendous financial losses in the pork industry, emphasizing the importance of disease resilience, which is the ability of an animal to maintain performance under disease. Previously, a natural polymicrobial disease challenge model was established, in which pigs were challenged in the late nursery phase by multiple pathogens to maximize expression of genetic differences in disease resilience. Genetic analysis found that performance traits in this model, including growth rate, feed and water intake, and carcass traits, as well as clinical disease phenotypes, were heritable and could be selected for to increase disease resilience of pigs. The objectives of the current study were to identify genomic regions that are associated with disease resilience in this model, using genome-wide association studies and fine-mapping methods, and to use gene set enrichment analyses to determine whether genomic regions associated with disease resilience are enriched for previously published quantitative trait loci, functional pathways, and differentially expressed genes subject to physiological states. Multiple quantitative trait loci were detected for all recorded performance and clinical disease traits. The major histocompatibility complex region was found to explain substantial genetic variance for multiple traits, including for growth rate in the late nursery (12.8%) and finisher (2.7%), for several clinical disease traits (up to 2.7%), and for several feeding and drinking traits (up to 4%). Further fine mapping identified 4 quantitative trait loci in the major histocompatibility complex region for growth rate in the late nursery that spanned the subregions for class I, II, and III, with 1 single-nucleotide polymorphism in the major histocompatibility complex class I subregion capturing the largest effects, explaining 0.8–27.1% of genetic variance for growth rate and for multiple clinical disease traits. This single-nucleotide polymorphism was located in the enhancer of *TRIM39* gene, which is involved in innate immune response. The major histocompatibility complex region was pleiotropic for growth rate in the late nursery and finisher, and for treatment and mortality rates. Growth rate in the late nursery showed strong negative genetic correlations in the major histocompatibility complex region with treatment or mortality rates (−0.62 to −0.85) and a strong positive genetic correlation with growth rate in the finisher (0.79). Gene set enrichment analyses found genomic regions associated with resilience phenotypes to be enriched for previously identified disease susceptibility and immune capacity quantitative trait loci, for genes that were differentially expressed following bacterial or virus infection and immune response, and for gene ontology terms related to immune and inflammatory response. In conclusion, the major histocompatibility complex and other quantitative trait loci that harbor immune-related genes were identified to be associated with disease resilience traits in a large-scale natural polymicrobial disease challenge. The major histocompatibility complex region was pleiotropic for growth rate under challenge and for clinical disease traits. Four quantitative trait loci were identified across the class I, II, and III subregions of the major histocompatibility complex for nursery growth rate under challenge, with 1 single-nucleotide polymorphism in the major histocompatibility complex class I subregion capturing the largest effects. The major histocompatibility complex and other quantitative trait loci identified play an important role in host response to infectious diseases and can be incorporated in selection to improve disease resilience, in particular the identified single-nucleotide polymorphism in the major histocompatibility complex class I subregion.

## Introduction

Pork is the most consumed animal protein in the world, accounting for about 32% of total animal protein consumption (UNEP 2012), while the demand for animal protein continues to grow. However, the pork industry faces unprecedented challenges from infectious diseases such as porcine reproductive and respiratory syndrome virus (PRRSV), porcine epidemic diarrhea virus, porcine circovirus (PCV), swine influenza, brachyspira-colitis (swine dysentery), *Streptococcus suis* septicemia, and others (VanderWaal and Deen 2018). These diseases cause tremendous financial losses in the pork industry, as well as reductions in animal welfare and increased use of antibiotics. Additionally, intense genetic selection for productivity has placed pressure on behavioral, physiological, reproductive, and immunological functions, impacting the ability of pigs to cope with disease challenges (Rauw et al. 1998; Prunier et al. 2010; Knap 2012). This emphasizes the importance of disease resilience, which is the ability of an animal to maintain performance upon exposure to a pathogen (Albers et al. 1987). Disease resilience captures both resistance and tolerance (Bisset and Morris 1996; Doeschl-Wilson et al. 2012), where disease resistance is defined as the ability of an animal to prevent infection when exposed to a pathogen or to limit replication of the pathogen when infected (Bishop and Stear 2003; Bishop 2012), and disease tolerance is defined as the ability of an animal to maintain performance at a given level of infection or pathogen load (Bishop 2012). One advantage of disease resilience is that it does not require knowledge of pathogen burden (Mulder and Rashidi 2017).

Multiple studies have been conducted to explore opportunities to select pigs for disease resilience (Albers et al. 1987; Bisset and Morris 1996; Doeschl-Wilson et al. 2012; Herrero-Medrano et al. 2015; Berghof et al. 2019; Poppe et al. 2020). However, in most of these studies, pigs were not challenged or challenged by only 1 pathogen. Putz et al. (2019) and Cheng et al. (2020) described the establishment of a natural polymicrobial disease challenge, in which late nursery pigs are challenged by multiple pathogens, including multiple viruses and bacteria, in order to maximize expression of genetic differences in disease resilience. Results demonstrated that the performance and clinical disease phenotypes recorded under this severe disease challenge environment were heritable and, therefore, can be improved by genetic selection (Putz et al. 2019; Cheng et al. 2020).

One of the genomic regions that has been identified in multiple studies to be important in host response to infectious disease in pigs is the major histocompatibility complex (MHC) or swine leukocyte antigen region. The MHC in pigs is among the most well-characterized MHC systems in nonhuman animal species (Hammer et al. 2020). Serão et al. (2014) reported the MHC region to account for ∼25% of the genetic variance for the level of PRRSV IgG in blood, which was reported as sample-to-positive ratio (S/P ratio) for a sow herd that experienced a PRRS outbreak. Sanglard et al. (2020) found the MHC region to explain 30% of the genetic variance for S/P ratio of PRRSV IgG in blood of sows following PRRSV vaccination in a PRRS-negative commercial farm. Walker et al. (2018) found that 2 single-nucleotide polymorphisms (SNPs) in the MHC class II subregion explained 3.8% of the genetic variation for PCV2b viral load in blood for the first 28 days following experimental infection of 5-week old pigs.

Using data generated in the natural disease challenge model described by Putz et al. (2019) and Cheng et al. (2020), the objectives of this study were (1) to identify genomic regions that are associated with disease resilience using genome-wide association studies (GWAS); (2) to evaluate whether genomic regions associated with disease resilience are enriched for previously published quantitative trait loci (QTL), functional pathways, and physiological states based on gene set enrichment analyses (GSEAs); (3) fine map the QTL in the MHC region that were identified under objective (1).

## Methods

### Ethical statement

This study was carried out in accordance with the Canadian Council on Animal Care guidelines (CCAC; https://www.ccac.ca/en/certification/about-certification, last accessed 12/30/2021). The protocol was approved by the Protection Committee of the Centre de Recherche en Sciences Animales de Deschambault (CRSAD) and the Animal Care and Use Committee at the University of Alberta (AUP00002227). The project was fully overseen by the Centre de développement du porc du Québec (CDPQ) in Québec, Canada, and its herd veterinarian together with project veterinarians.

### Natural challenge protocol

**Fig. 1.**
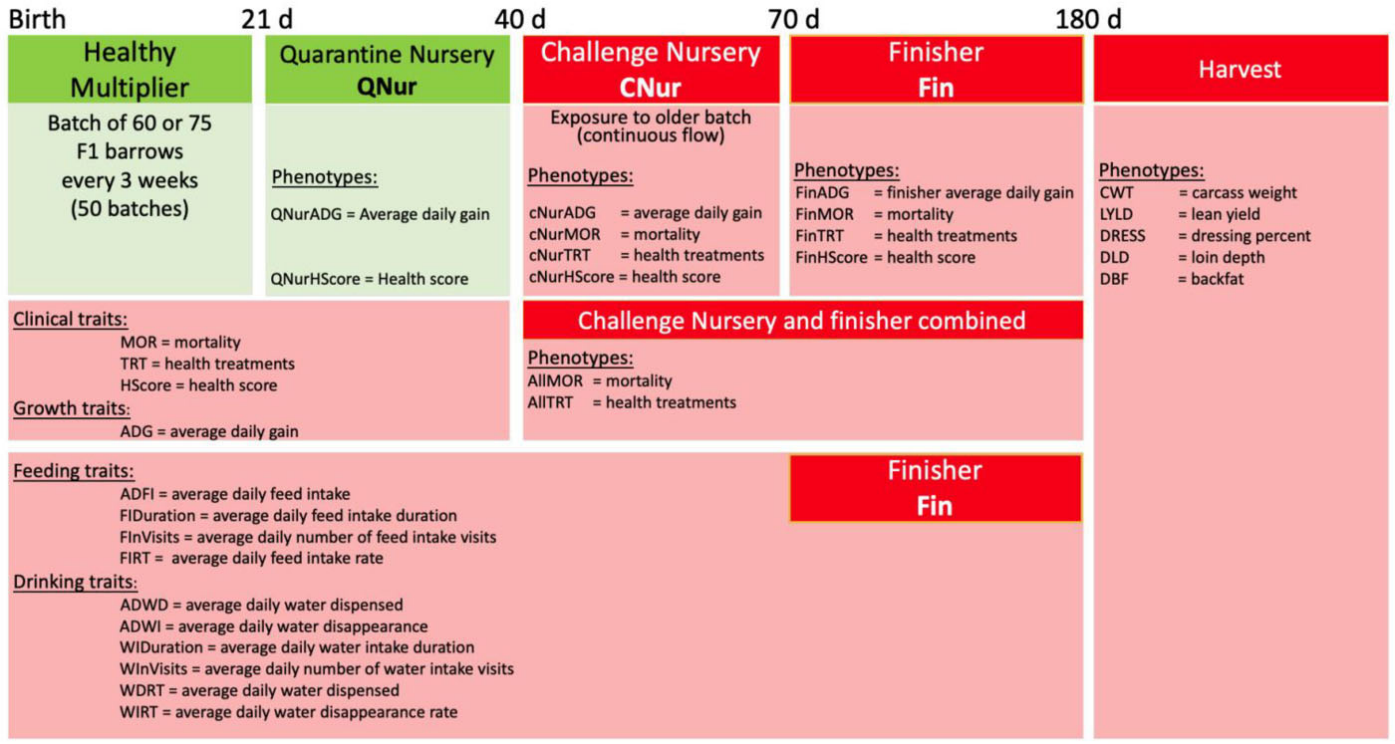
Natural polymicrobial disease challenge protocol and recorded phenotypes and their abbreviations. Green, healthy; red, challenged.

A natural disease challenge wean-to-finish protocol (Fig. 1) was established in 2015 by bringing naturally infected animals into a nursery and finish research facility at CDPQ, targeting various viral and bacterial diseases, and maintained by entering batches of 60–75 healthy nursery pigs every 3 weeks in a continuous flow system (see Putz et al. 2019, for details). The natural challenge protocol consisted of 3 phases: (1) quarantine nursery (19 days on average, beginning at 3 week of age); (2) challenge nursery (27 days on average); and (3) finishing phase (100 days on average). The average group sizes in the 3 phases were 4.25, 7.16, and 10.72 pigs per pen, respectively. Pigs were re-grouped when moved to the challenge nursery and to the finisher. A fixed weight system was used to identify pigs for slaughter every 3 weeks, with pigs that had reached the target weight of 135 kg sent for slaughter.

### Phenotypes

A broad spectrum of disease resilience phenotypes was collected on each pig, including growth performance in each phase, clinical disease traits (medical treatment rates, mortality rates, and subjective health scores) in the challenge nursery and finisher, carcass traits, and feed intake and water intake and behavior traits in the finisher. Data on 3,285 Large White by Landrace barrows from 7 breeding companies were available, as described by Cheng et al. (2020, 2021). All data and samples were collected by trained research staff from CDPQ using established natural challenge protocols. Body weights were obtained on all pigs at entry and exit of each phase and every 3 weeks in the finisher. Individual feed intake data were recorded in the finishing barn using IVOG feeding stations (Insentec, Marknesse, the Netherlands) and edited using the methods of Casey et al. (2005). Individual water intake data were also recorded in the finishing barn using a custom-made individual water intake recording system for each pen and edited using the method developed by Cheng et al. (2021).

Details of the phenotypes recorded and analyzed in the 3 phases are in Cheng et al. (2020, 2021). Subjective health scores based on clinical signs were assigned by trained personnel in the quarantine nursery (qNurHScore), the challenge nursery (cNurHScore), and the finisher (FinHScore). Health scores were on a 1–5 scale: 1 = severe clinical signs with wasting and 5 = in perfect health, as described by Cheng et al. (2020). Because of limited numbers of animals receiving low scores, scores ≤4 were assigned a score of 4 for qNurHscore, and scores ≤3 were assigned a score of 3 for cNurHScore and FinHScore. Treatment rates were adjusted by multiplying the number of treatments a pig received in the corresponding phase by the ratio of the average length of the phase and the number of days the pig spent in the phase and included challenge nursery treatment rate (cNurTRT), finisher treatment rate (FinTRT), and treatment rate across the challenge nursery and finisher (AllTRT). Mortality was recorded as 0 = survived and 1 = died in the challenge nursery (cNurMOR), the finisher (FinMOR), and across the challenge nursery and finisher (AllMOR). Performance traits included average daily gain (ADG) in the quarantine nursery (qNurADG), the challenge nursery (cNurADG), and the finisher (FinADG), and the slaughter traits carcass weight (CWT), dressing % (DRESS), lean yield (LYLD), carcass backfat (DBF), and carcass loin depth (DLD). ADG was computed by Cheng et al. (2020) and carcass traits were calculated following Putz et al. (2019). Feeding and drinking traits were computed following Cheng et al. (2020, 2021) and included average daily feed intake (ADFI), ADFI duration (FIDuration), average daily number of feed intake visits (FInVisits), ADFI rate (FIRT), average daily water dispensed (ADWD), average daily water disappearance (ADWI), average daily water intake duration (WIDuration), average daily number of water intake visits (WInVisits), ADWD rate (WDRT), and ADWI rate (WIRT). For treatment and growth rates and feeding traits in the finisher, data from pigs that died in the finisher were included in the analyses, with imputation and expansion of treatment and growth rates, as described in Cheng et al. (2020).

### Genotypes

All animals were genotyped with the 650k Affymetrix Axiom Porcine Genotyping Array by Delta Genomics (Edmonton AB, Canada). In total, 658,692 SNPs were included on the chip. The 435,172 SNPs that passed quality control for all 7 cycles, as described by Cheng et al. (2020), were utilized for analysis.

### Genome-wide association analyses

The following marker-based Bayes-B model (Habier et al. 2011) was used for GWAS:

$$y_{\mathrm{ijkl}}=\mathrm{Batc}h_{i}+D{\mathrm{ied}}_{\mathrm{ijkl}}+\;b\times \mathrm{Ag}e_{\mathrm{ijkl}}+\;\mathrm{Pe}n_{j}+\;\mathrm{litte}r_{\mathrm{ijk}}+\sum_{n=1}^{p}m_{\mathrm{ijkln}}\beta_{n}\delta_{n}+\;e_{\mathrm{ijkl}}$$

where $y_{\mathrm{ijkl}}$ is the phenotype of pig *ijkl*; $\mathrm{Batc}h_{i}$ is a fixed batch effect (*i* = 1, …, 50); $\mathrm{Ag}e_{\mathrm{ijkl}}$ is the covariate of age when the pig entered the quarantine nursery; $D{\mathrm{ied}}_{\mathrm{ijkl}}$ is the fixed effect of the pig dying during the corresponding phase, which was fitted only for cNurADG, FinADG, FinTRT, ADFI, FIDuration, and AllTRT; $\mathrm{Pe}n_{j}$ is the random effect of pen within batch for the corresponding phase; $\mathrm{Pe}n_{j}\;∼\;N(0,\;\sigma_{P}^{2})$, where $\sigma_{P}^{2}$ is the pen variance; $\mathrm{litte}r_{\mathrm{ijk}}$ is the litter environmental effect; $\mathrm{litte}r_{\mathrm{ijk}}∼\;N(0,\;\sigma_{l}^{2}$), where $\sigma_{l}^{2}$ is the litter environmental variance; $m_{\mathrm{ijkln}}$ is genotype for SNP *n* (coded as 0, 1, and 2), with allele substitution effect $\beta_{n}\delta_{n}$, where $\delta_{n}$ is an indicator whether SNP *n* was included ($\delta_{n}$ = 1) or excluded ($\delta_{n}$ = 0) in the model, with a prior probability of 0.1% of an SNP having a nonzero effect (π=0.999); $\beta_{n}\;∼\;N(0,\;\sigma_{n}^{2})$, where $\sigma_{n}^{2}$ is the locus-specific marker effect variance that was assumed to follow a scaled inverted chi-square distribution with scale parameter of $S_{\beta }$ and degrees of freedom of $v_{\beta }$; p is the number of genotyped SNPs; and $e_{\mathrm{ijkl}}$ is the residual effect, with $e_{\mathrm{ijkl}}∼\;N(0,\;\sigma_{e}^{2}$), where $\sigma_{e}^{2}$ is the residual variance. Mortality was analyzed as a binary trait using a Bayes-B threshold model (Sorensen et al. 1995).

Bivariate analyses were performed using the same model as used for the univariate analyses, following Cheng et al. (2018a). For these analyses, mortality was analyzed as a continuous phenotype. Random pen, litter, and residual effects for the 2 analyzed traits were assumed distributed multivariate normal, with $R_{i}$**∼ MVN** (0,$\;U_{i}$), where $U_{i}$ = $\left[\begin{array}{c} \begin{array}{cc} {\sigma_{U_{i}}}_{1}^{2} & {\sigma_{U_{i}}}_{1,2}\\ {\sigma_{U_{i}}}_{2,1} & {\sigma_{U_{i}}}_{2}^{2} \end{array} \end{array}\right],$ with *i* representing the random effects of pen, litter, and residuals, and $U_{i}$ assumed to have an inverse Wishart prior distribution, $W_{t}^{-1}(S_{i},\;v_{i})$, where *t* = 2, $S_{i}$ and $v_{i}$ are the scale parameter and the degree of freedom for the random effect. Allele substitution effects for the 2 traits at a particular SNP can be written as $D_{n}\beta_{n}$, where $\beta_{n}$**∼ MVN**(0, ***G_n_***), where ***G_n_*** is a locus-specific covariance matrix = $\left[\begin{array}{c} \begin{array}{cc} {{\sigma_{\beta }}_{n}}_{1}^{2} & {{\sigma_{\beta }}_{n}}_{1,2}\\ {{\sigma_{\beta }}_{n}}_{2,1} & {{\sigma_{\beta }}_{n}}_{2}^{2} \end{array} \end{array}\right]$, a priori assumed to follow an inverse Wishart distribution, $W_{t}^{-1}(S_{\beta }+\beta_{n}\beta_{n}^{'},\;v_{\beta }+1)$, where *t* is the number of traits with *t* = 2, $S_{\beta }$ is the scale parameter and $v_{\beta }$ is the degree of freedom, and $D_{n}=\;\left[\begin{array}{cc} \delta_{n1} & 0\\ 0 & \delta_{n2} \end{array}\right]$, where $\delta_{n1},\;\delta_{n2}$ are indicators for inclusion of nonzero effects of SNP *n* for trait 1 and 2, resulting in 4 combinations for $D_{n}$: (0, 0), (1, 0), (0, 1), and (1, 1), with assumed prior probabilities equal to 0.999×0.999, $0.999\times \left(1-0.999\right)$, $\left(1-0.999\right)\times 0.999$, and $\left(1-0.999\right)\times \left(1-0.999\right)$, respectively.

Univariate and bivariate GWAS were implemented using the JWAS package (Cheng et al. 2018b) using a Monte Carlo Markov chain of length 50,000, with the first 5,000 iterations discarded as burn-in. Allele substitution effects were estimated as the posterior mean of saved samples at every 100th iteration of the chain, while the posterior probability of inclusion of each marker was calculated as the proportion of iterations the marker was included in the model with a nonzero effect ($\delta_{i}$=1). Samples of the breeding value of each individual at each saved iteration were calculated as the sum of its SNP genotypes multiplied by the sampled marker effects for that iteration. For bivariate analyses, the genetic correlation between the 2 analyzed traits for a genomic region or across the whole genome was estimated as the posterior mean of the correlation across animals between the sampled breeding values for the 2 traits in the genomic region or across the whole genome, and its standard deviation (SD) across saved iterations as the standard error (SE). The percentage of genetic covariance captured by a genomic region was computed for each saved iteration as the ratio of the covariance of sampled breeding values for the region between the 2 traits to the covariance of sampled breeding values for the whole genome. The saved samples were also used to estimate the posterior distribution for genetic variance explained by each 1 or 0.25-Mb nonoverlapping window of the reference genome (Sus scrofa 11.1), which was used to estimate window-based posterior probabilities of association (WPPA Fernando et al. 2017), which is the posterior probability that at least 1 SNP in the window has a nonzero marker effect, with a high WPPA suggesting a high probability of presence of a QTL in this window.

Genomic regions that harbor a QTL were identified based on results of the univariate analyses by first identifying 1-Mb windows that explained more than 1% of the genetic variance and combining neighboring “significant” 1-Mb windows to constitute a QTL region. For the bivariate analyses, to identify genomic regions that were “significant” for both analyzed traits, initially 1-Mb windows that explained more than 1% of the genetic variance for both traits were used as criterion. However, this resulted in very few significant windows, even when the threshold was reduced to 0.5%. This approach also missed genomic regions that were significant for both traits but in neighboring 1-Mb windows. To address this, a sliding 3-Mb window was used, requiring the window to explain at least 0.75% of genetic variance for both traits.

### Fine-mapping haplotype model for the MHC region

Univariate Bayesian Interval Mapping (BayesIM), as described in Kachman (2015) and Wilson-Wells and Kachman (2016), was implemented for cNurADG, AllTRT, AllMOR, WIDuration, and WInVisits to derive haplotype effects across the genome for the purposes of fine mapping, with a focus on the MHC region. The same statistical model as described for the SNP-based GWAS was used here, except that haplotype effects were fitted instead of marker effects. A hidden Markov model based on Scheet and Stephens (2006) was used to generate 8 haplotype clusters based on SNP genotypes around each putative QTL, which were positioned every 5 kb across the genome. The average haplotype size (average distance between cluster recombination events) was set to 500 kb. Genetic variances, haplotype effects, and model frequencies at each QTL were estimated using a hierarchical Bayes-C model (Habier et al. 2011), with prior probability of a QTL having a nonzero effect set equal to 0.1%. The length of the Monte Carlo Markov Chain was 50,000, with the first 5,000 used for burn-in. If a QTL was included in the model, its haplotype effects for each cluster were modeled as independent normal random variables. Although the whole genome was analyzed, the focus here was on the MHC region. To identify QTL regions, first, QTL that explained more than 0.0005% of the genetic variance were filtered and neighboring QTL that also met this criterion were merged into QTL regions, allowing the region to include isolated QTL that did not meet this criterion but that were flanked by QTL that did. Then, if the combined QTL within a QTL region explained more than 0.1% of the genetic variance, the region was counted as a detected QTL region.

### Gene set enrichment analysis

To identify candidate genes, each QTL region that was identified in the univariate SNP-based GWAS was expanded by 1 additional megabase on either side of the QTL region. Genes located in each expanded QTL region were identified in BioMart (Smedley et al. 2009), using the ENSEMBL pig gene database (Sscrofa11.1). GSEA were conducted using the GSEA_4.0.3 software (Subramanian et al. 2005) based on 3 customized libraries that were derived based on (1) published QTL for pigs, (2) pig transcriptome information, and (3) the gene ontology (GO) database. The pig QTL library was created using the Animal QTL database (QTLdb) developed by Hu et al. (2007), which houses all publicly available QTL and SNP or gene association data on livestock species. For the pig, the QTLdb (accessed 2020 August 1) included 30,869 QTL from 706 publications for 692 different traits. These QTL were categorized into 107 trait types, such as immune capacity, disease susceptibility, growth, anatomy, etc. The transcriptome library was adapted from the Porcine Signature Database (PorSignDB; Van Renne et al. 2018), which is a collection of annotated gene sets for use with the GSEA software. These gene sets were mostly derived from in vivo derived transcriptomic data and describe a wide spectrum of (patho)physiological states of different porcine tissue types. The GO database was adapted from the Molecular Signatures Database (MSigDB, c5.all.v7.0.symbols.gmt; Subramanian et al. 2005), which provides a large collection of curated gene sets. Using these 3 data sources, 3 GSEA libraries were created by assigning their respective features to each nonoverlapping 0.25-Mb window across the genome (9,058 windows in total). Features that were assigned to fewer than 11 or to more than 8,999 windows were removed. Then, GSEA was conducted separately for each of the 3 libraries (QTL, transcriptome, and GO) for each resilience trait, except for feeding and drinking traits, using a list of 0.25-Mb windows that was ranked by the % of genetic variance explained by the 0.25-Mb window based on the univariate SNP-based GWAS analyses, with the following options: number of permutations = 1,000; collapse/remap to gene symbol = no collapse; enrichment statistics = weighted.

## Results

### Univariate GWAS to detect QTL

**Fig. 2.**
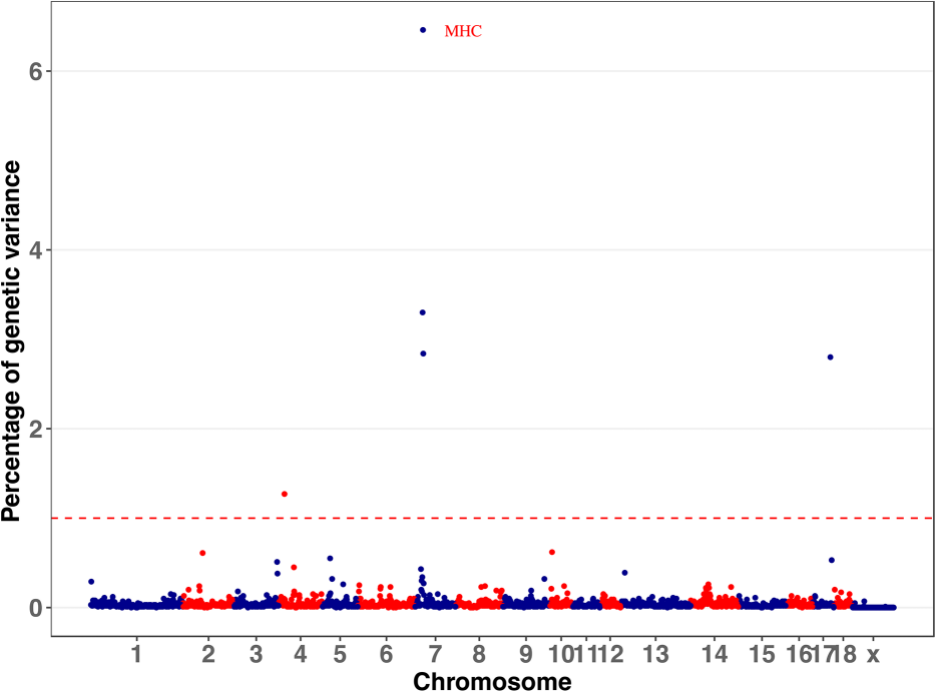
Percentage of genetic variance explained by nonoverlapping 1-Mb windows across the genome for ADG in the challenge nursery based on the univariate Bayes-B analysis. Windows that explained more than 1% of genetic variance (broken line) were considered significant. MHC, major histocompatibility complex.

Genomic regions associated with each trait were identified using univariate GWAS based on variable selection method Bayes-B (Habier et al. 2011) by identifying nonoverlapping 1-Mb windows that were estimated to explain more than 1% of the genetic variance for the trait analyzed. These genomic regions were combined with any flanking “significant” 1-Mb windows to constitute a QTL region. QTL regions identified are shown in Table 1 and Supplementary Figs. 1–9, and estimates of trait heritabilities based on the GWAS are in Table 2. For most traits, estimates of heritability based on GWAS were close or slightly lower than estimates for the same data based on genomic relationships by Cheng et al. (2020, 2021) (Supplementary Fig. 10). Across traits, the GWAS identified 59 QTL regions, which on average each explained 2.2% of the genetic variance, with a maximum of 12.6% for the MHC region for cNurADG (Fig. 2). The maximum total genetic variance explained by all identified QTL for a trait was 16.7%, for cNurADG, and the maximum number of QTL detected for a trait was 7, for DBF. In general, more QTL were detected for traits that were more heritable (Supplementary Fig. 10), except for WinVisits and WDRT, which had high heritabilities but only 1 QTL detected each. Most QTL had moderate to high WPPA (0.18–1). The WPPA was 1 for some QTL for LYLD, DBF, and WIDuration (Table 1).

**Table 1.** QTL detected (>1% of genetic variance) in nonoverlapping 1-Mb windows based on the univariate Bayes-B analyses, along with positional candidate genes and their function.

SSC	Position (Mb)	Trait	% of genetic variance	WPPA	Candidate genes	Gene function
1	7	FinMOR	2.10	0.18	*IGF2R*, *TAGAP*	Immune system regulation, T-cell activation
		AllMOR	4.24	0.60		
1	22	ADWD	1.67	0.80		
		WDRT	2.31	0.70		
1	150	FinADG	1.20	0.40		
1	161	DBF	1.44	0.75		
2	1	LYLD	10.48	1.00	*IGF2*, *CD81*, *LSP1*, *IFITM1*	Muscle mass and fat deposition, T-cell recognition and activation, neutrophil motility regulation, immune response signaling
		DBF	6.61	1.00		
2	41	DBF	1.09	0.71		
2	119	ADWI	1.01	0.52	*TICAM2*	Adaptive immune response
3	18	ADWI	1.08	0.63		
3	27	DLD	1.17	0.50		
3	129	cNurTRT	2.68	0.73		
		FinADG	1.04	0.56		
		DBF	1.73	0.90	*RSAD2*	Virus inhibition
		ADFI	1.10	0.59		
4	17	cNurADG	1.27	0.52		
4	41	FinADG	1.53	0.34		
4	44	FinADG	1.36	0.46		
4	56	FInVisits	2.85	0.98	*IL-7*	B and T-cell development
4	84	WIDuration	1.15	0.73	*CD247*, *ILDR2*	Immune response
4	95	ADWI	1.14	0.68	*IL6R*	Immune system regulation
5	66	qNurADG	1.18	0.56		
5	69	cNurHScore	1.44	0.55	*IL17RA*	Bacterial and fungal infection defense
5	85	LYLD	1.07	0.72		
6	86	LYLD	1.61	0.81	*DNAJC8*	Heat shock protein
6	102	qNurHscore	1.68	0.71		
6	141	DLD	1.01	0.52		
6	147	DBF	1.09	0.58	*DNAJC6*	Heat shock protein
6	162	WIRT	1.58	0.68		
6	165	LYLD	1.52	0.96	*MKNK1*	Response to environmental stress
7	4	FIRT	1.56	0.70	*LY86*	Innate immune response
7	22	FinADG	1.44	0.57	*MHC Class I*	MHC QTL22
		ADFI	2.79	0.86		
		FIRT	1.25	0.53		
7	22–24	cNurADG	12.6	0.67–0.89	*MHC Class I*, *II*, and *III*	MHC QTL22, QTL23, QTL24
7	23	ADWD	1.72	0.68	*MHC Class I and III*	MHC QTL23
7	25	WIDuration	3.58	1.00	*MHC Class II*	MHC QTL25
		WInVisits	1.51	0.70		
7	26	cNurHScore	1.23	0.46		
7	40	DRESS	1.19	0.33	*HSP90AB1*	Heat shock protein
7	63	ADWD	1.37	0.76	*NFKBIA*	Immune and proinflammatory response
8	28	DLD	1.03	0.39		
8	117	WDRT	1.32	0.58		
8	124	DBF	1.66	0.76		
8	131	AllTRT	1.64	0.42		
8	138	WIRT	1.41	0.48		
9	31	ADFI	1.15	0.50		
9	104	WIDuration	2.14	0.83	*DNAJC2*	Heat shock protein
10	40	qNurADG	2.02	0.83	*MAP3K8*	Innate immune response
11	67	ADWD	1.15	0.63		
12	41	ADFI	1.20	0.65	*CCL1*, *2*, *8*, and *11*	Immunoregulatory and inflammatory process
13	64	CWT	1.38	0.48		
		DRESS	1.29	0.45		
13	134–135	qNurADG	7.21	0.39–0.73	*NRROS*	Bacterial and fungal infection defense
13	154	ADWD	4.58	0.88	*ALCAM*	T-cell activation and proliferation
13	178	qNurHscore	1.02	0.53	*HSPA13*	Heat shock protein
14	23	AllTRT	5.11	0.85	*ULK1*	Interferon-dependent immunity
15	127	FinHScore	1.17	0.34		
16	0	qNurHscore	2.64	0.83		
16	3	FinADG	1.11	0.59		
16	5	CWT	1.10	0.41		
		DRESS	1.40	0.45		
16	51–52	ADFI	2.95	0.43–0.44	*TLX3*, *LCP2*	T-cell activation and proliferation
16	74	FInVisits	1.12	0.60		
17	14	qNurHscore	5.83	0.32		
17	26	FIDuration	1.76	0.81		
17	32	LYLD	1.32	0.86	*MAVS*, *HSPA12B*	Antiviral innate immunity, heat shock protein
		DBF	2.26	0.94		
17	53	cNurADG	2.80	0.82	*IL-29*, *NFATC2*	Immune response
		FIDuration	1.78	0.85		
18	47	ADWI	2.94	0.86		

SSC, Sus scrofa chromosome; Pos, genomic position; gVar, genetic variance; WPPA, window-based posterior probability of association; qNurADG, average daily gain in the quarantine nursery; qNurHScore, health score in the quarantine nursery; cNurADG, average daily gain in the challenge nursery; cNurHScore, health score in the challenge nursery; cNurTRT, treatment rate in the challenge nursery; cNurMOR, mortality rate in the challenge nursery; FinADG, average daily gain in the finisher; FinHScore, health score in the finisher; FinTRT, treatment rate in the finisher; FinMOR, mortality rate in the finisher; AllTRT, treatment rate across the challenge nursery and finisher; AllMOR, mortality rate across the challenge nursery and finisher; CWT, carcass weight; DRESS, dressing percent; LYLD, lean yield; DBF, backfat; DLD, loin depth; ADFI, average daily feed intake; FIDuration, average daily feed intake duration; FInVisits, average daily number of feed intake visits; FIRT, average daily feed intake rate; ADWD, average daily water dispensed; ADWI, average daily water disappearance; WIDuration, average daily water intake duration; WInVisits, average daily number of water intake visits; WDRT, average daily water dispensed; WIRT, average daily water disappearance rate. MHC QTL22, QTL23, QTL24, and QTL25 were identified for cNurADG as shown in Fig. 7.

**Table 2.** Estimates of the effect, the percentage of genetic variance explained by the SNP and the expanded MHC region, and marker-based heritability based on the ASReml analyses with SNP AX-116313535 fitted as fixed effect.

Trait	Estimates of the effects of SNP AX-116313535 (SE)			% genetic variance by		Heritability
	Additive effect	Dominance effect	Allele substitution effect	SNP	MHC	
Quarantine nursery						
ADG, kg/d	0.001 (0.003)	0	−0.001 (0.002)	0	1.4	0.28
Health score	0.006 (0.01)	−0.009 (0.02)	0.0008 (0.01)	0	0.7	0.18
Challenge nursery						
ADG, kg/d	0.023 (0.006)	−0.005 (0.006)	−0.026 (0.004)	9.4	12.8	0.19
Treatment rate	0.123 (0.05)	−0.027 (0.06)	0.108 (0.04)	2.6	1.2	0.11
Mortality rate	0.037 (0.01)	−0.037 (0.02)	0.016 (0.01)	1.6	0.6	0.05
Health score	0.075 (0.03)	0.040 (0.03)	−0.052 (0.02)	2.1	2.7	0.13
Finisher						
ADG, kg/d	0.012 (0.006)	0.005 (0.007)	−0.009 (0.005)	0.8	2.7	0.24
Treatment rate	0.090 (0.03)	−0.009 (0.04)	0.085 (0.02)	27.1	0.7	0.04
Mortality rate	0.018 (0.02)	0.009 (0.02)	0.023 (0.01)	3.2	0.5	0.04
Health score	0.092 (0.03)	0.044 (0.03)	−0.068 (0.02)	9.4	1.3	0.07
cNursery and finisher combined						
Treatment rate	0.153 (0.06)	−0.015 (0.07)	0.145 (0.04)	4.3	2.2	0.10
Mortality rate	0.047 (0.02)	−0.025 (0.02)	0.033 (0.01)	2.6	1.1	0.12
Carcass						
LYLD	0.008 (0.08)	−0.075 (0.09)	−0.032 (0.06)	0	1.0	0.32
DBF	0.056 (0.17)	0.107 (0.19)	0.001 (0.13)	0	1.3	0.41
DLD	0.336 (0.28)	−0.289 (0.33)	−0.490 (0.23)	0.9	0.5	0.37
CWT	0.065 (0.12)	−0.252 (0.14)	−0.073 (0.09)	0.3	0.7	0.24
DRESS	0.094 (0.10)	−0.205 (0.11)	−0.018 (0.07)	0	0.6	0.23
Feed and water intake and behavior traits						
ADFI, kg/d	0.029 (0.01)	0.009 (0.02)	−0.024 (0.01)	0.8	3.7	0.29
FIDuration, min/d	1.098 (0.50)	0.806 (0.57)	−0.666 (0.40)	0.3	0.6	0.35
FInVisits	0.284 (0.30)	0.082 (0.34)	−0.240 (0.24)	0.1	1.1	0.32
FIRT	0	0	−0.0001 (0.0003)	0	2.0	0.24
ADWI, l/d	0.150 (0.13)	0.053 (0.15)	−0.122 (0.10)	0.4	0.8	0.41
ADWD, l/d	0.274 (0.19)	0.026 (0.22)	−0.260 (0.15)	0.6	3.2	0.37
WIDuration, min/d	0.077 (0.21)	−0.247 (0.24)	−0.208 (0.17)	0.2	4.0	0.39
WInVisits	0.248 (0.36)	−0.042 (0.41)	−0.270 (0.29)	0.1	2.3	0.42
WIRT	0.003 (0.007)	−0.002 (0.009)	−0.004 (0.006)	0.2	0.5	0.31
WDRT	0.011 (0.01)	−0.004 (0.01)	−0.014 (0.008)	0.7	1.2	0.28

Genetic variance explained by this SNP was computed as 2pq$\alpha^{2}$, where α is the allele substitution effect, *P* = 0.25 and *q* = 0.75, are the allele frequencies for A and G, respectively, AA coded as 0, AG coded as 1, and GG coded as 2.

gVar, genetic variance; SNP, single-nucleotide polymorphism; MHC, major histocompatibility complex; ADG, average daily gain; CWT, carcass weight; DRESS, dressing percent; LYLD, lean yield; DBF, backfat; DLD, loin depth; ADFI, average daily feed intake; FIDuration: average daily feed intake duration; FInVisits: average daily number of feed intake visits; FIRT, average daily feed intake rate; ADWD, average daily water dispensed; ADWI, average daily water disappearance; WIDuration, average daily water intake duration; WInVisits, average daily number of water intake visits; WDRT, average daily water dispensed; WIRT, average daily water disappearance rate. Heritability was estimated as the ratio of genetic variance to the phenotypic variance.

Some QTL regions were significant for multiple traits (Table 1), suggesting presence of pleiotropic QTL. A 1-Mb region at 1 Mb on SSC2 was identified for both LYLD and DBF. The region at 129 Mb on SSC3 was identified for 4 traits, i.e. cNurTRT, FinADG, DBF, and ADFI. The window at 53 Mb on SSC17 was detected for cNurADG and for FIDuration. The MHC region was detected as a QTL region for 7 traits, i.e. cNurADG, FinADG, ADFI, FIRT, ADWD, WIDuration, and WInVisits.

**Fig. 3.**
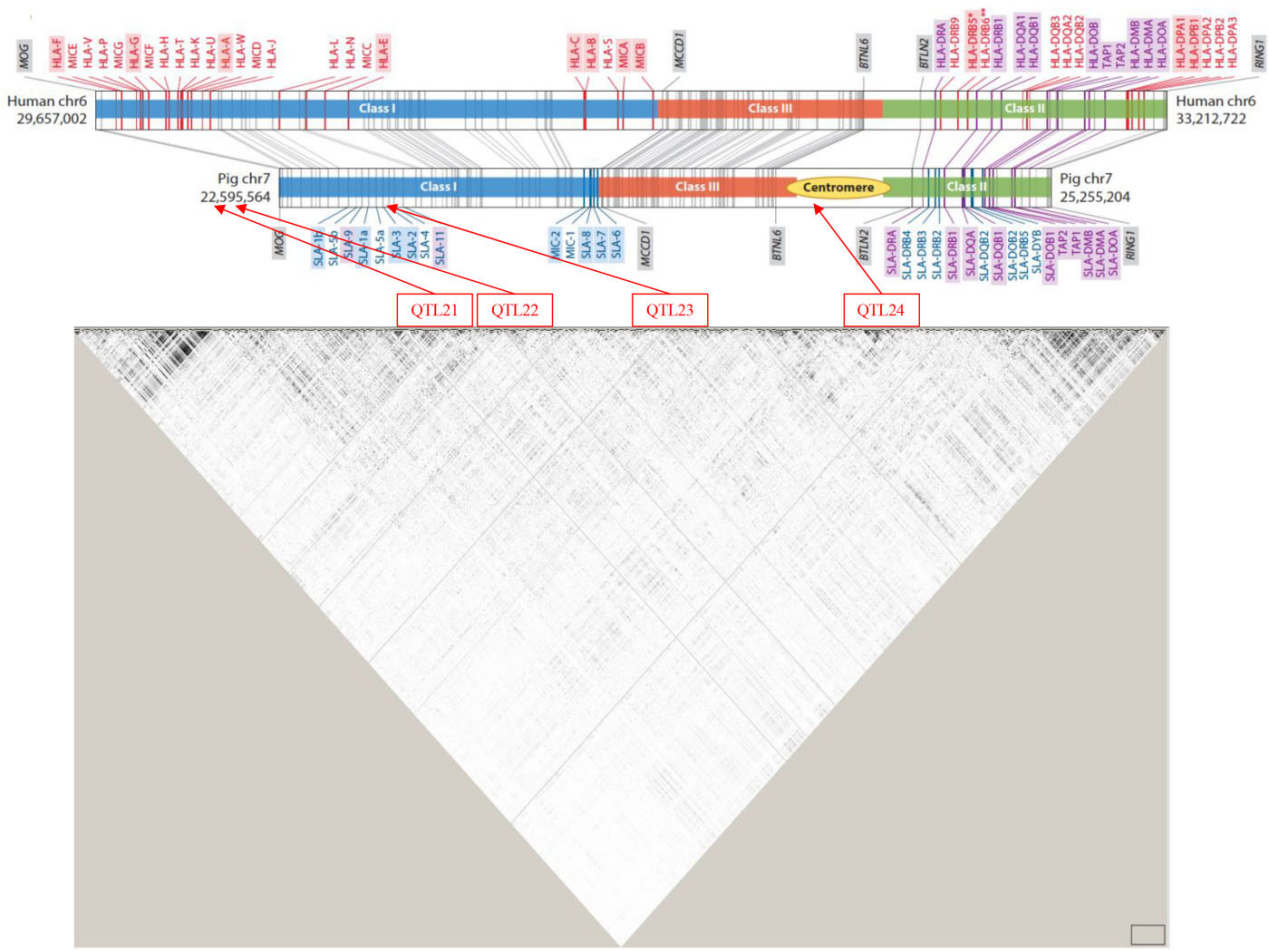
LD (r^2^) in the MHC region based on the Haploview software (Barrett et al. 2005), with location of the identified QTL for ADG in the challenge nursery based on the Bayes-IM analysis relative to the annotated map of the MHC in the pig and human (from Hammer et al. 2020). Modified with permission from the Annual Review of Animal Biosciences, Volume 8 © 2020 by Annual Reviews, http://www.annualreviews.org.

For further analyses, the MHC region was expanded to the 20–30-Mb region on SSC7, which will hereafter be referred to as the expanded MHC region, based on linkage disequilibrium (LD) (Fig. 3) and additional 1-Mb windows with substantial genetic variance that were identified on either side of the MHC region. The percentage of genetic variance explained by the expanded MHC region for each trait is provided in Table 2 and was >1% for several traits that did not reach 1% for the original MHC region.

### Bivariate GWAS to detect pleiotropic QTL

To further characterize pleiotropic QTL, genomic regions that were “significant” for 2 traits in the univariate analyses were further analyzed using a bivariate Bayes-B GWAS. Initially, the focus was on nonoverlapping 1-Mb windows that were estimated to explain more than 1% of the genetic variance for both traits analyzed but this resulted in very few significant windows, even when the threshold was reduced to 0.5%. This approach also missed genomic regions that were significant for both traits but for neighboring 1-Mb windows. Hence, a sliding 3-Mb window was used instead, along with a threshold of requiring at least 0.75% of the genetic variance to be explained for each of the 2 traits analyzed. Results are summarized in Table 3. The % genetic covariance for these 3-Mb regions was computed as the ratio of the estimated genetic covariance for the region to the estimated genetic covariance for the whole genome. In total, 18 pleiotropic QTL regions were detected, with genetic covariance ratio estimates ranging from −60% to 107%, noting that the genetic covariance estimated for a region can be larger than the genome-wide covariance or have an opposite sign. All identified pleiotropic regions involved at least 1 ADG trait.

**Table 3.** Pleiotropic QTL detected (>0.75% of genetic variance for both traits) based on 3-Mb windows in bivariate Bayes-B analyses and estimates of the genetic variance, covariance, and correlation for the QTL, along with positional candidate genes and their function.

SSC	Region (Mb)	Trait	% of genetic		Genetic correlation		Candidate genes	Gene function
			Variance	Covariance	Region	Genome		
2	149–151	FinADG	1.8	−1.3	−0.31 (0.64)	−0.05 (0.06)	*CD74*	Immune response
		FinHScore	1.0					
3	35–37	qNurADG	0.8	2.2	0.30 (0.64)	0.07 (0.06)	*DNAJA3*	Heat shock protein
		qNurHScore	0.8					
3	127–130	cNurADG	3.9	−1.3	−0.53 (0.43)	−0.33 (0.06)	*RSAD2*	Virus inhibition
		cNurMOR	0.8					
		cNurADG	4.6	−2.5	−0.75 (0.33)	−0.39 (0.06)		
		cNurTRT	4.3					
		cNurADG	3.1	−1.9	−0.67 (0.42)	−0.43 (0.06)		
		AllMOR	1.7					
		cNurADG	1.2	−10.3	−0.43 (0.56)	−0.12 (0.06)		
		AllTRT	1.9					
		FinADG	3.1	−1.7	−0.40 (0.55)	−0.11 (0.07)		
		AllTRT	1.3					
		FinADG	4.1	7.7	0.41 (0.52)	0.04 (0.06)		
		cNurADG	0.8					
4	43–46	FinADG	2.4	−37.0	−0.79 (0.42)	0.04 (0.06)		
		cNurADG	1.0					
4	101–103	qNurADG	3.2	−6.7	−0.48 (0.55)	0.07 (0.06)	*VTCN1*, *CD101*, *CD2*, *IGSF3*	T-cell mediated immune response
		cNurADG	1.8					
5	13–15	qNurADG	1.0	−2.0	−0.25 (0.62)	0.07 (0.06)	*CKAP4*, *DNAJC22*	Innate immune system
		qNurHScore	1.1					Heat shock protein
7	16–18	qNurADG	1.4	17.3	0.59 (0.45)	0.07 (0.06)		
		cNurADG	1.9					
7	21–25	cNurADG	14.5	−10.8	−0.62 (0.29)	−0.33 (0.06)	*MHC Class I*, *III*, and *II*	MHC
		cNurMOR	3.7					
		cNurADG	4.9	−12.8	−0.84 (0.19)	−0.39 (0.06)		
		cNurTRT	3.1					
		cNurADG	9.3	−20.0	−0.85 (0.15)	−0.43 (0.06)		
		AllMOR	8.7					
		cNurADG	15.7	−60.0	−0.70 (0.27)	−0.12 (0.06)		
		AllTRT	3.2					
		cNurADG	11.2	27.4	0.27 (0.32)	0.08 (0.06)		
		cNurHScore	1.1					
		FinADG	3.3	107.0	0.79 (0.42)	0.04 (0.06)		
		cNurADG	8.0					
7	92–94	FinADG	0.8	0.7	0.15 (0.56)	−0.11 (0.06)		
		AllMOR	1.0					
9	30–32	FinADG	1.3	−1.8	−0.50 (0.61)	−0.11 (0.07)		
		AllTRT	1.0					
10	39–41	qNurADG	1.4	16.3	0.58 (0.51)	0.07 (0.06)	*MAP3K8*	Innate immune response
		cNurADG	2.2					
11	21–23	qNurADG	3.1	4.6	0.48 (0.51)	0.07 (0.06)	*LCP1*	T-cell activation
		qNurHScore	1.5					
13	133–135	qNurADG	4.2	16.3	0.55 (0.58)	0.07 (0.06)	*NRROS*	Bacterial and fungal infection defense
		cNurADG	0.8					
14	46–48	cNurADG	1.0	−0.7	−0.74 (0.52)	−0.33 (0.06)	*LIF*	T-cell immunity
		cNurMOR	1.6					
15	0–1	qNurADG	1.1	0.50	0.56 (0.59)	0.07 (0.06)	*STAM2*	T-cell development
		qNurHScore	1.0					
15	113–115	cNurADG	0.9	−4.0	−0.53 (0.61)	0.08 (0.06)	*LANCL1*	Antimicrobial activity
		cNurHScore	0.8					
16	2–4	FinADG	1.1	−1.8	−0.37 (0.62)	−0.11 (0.07)	*OTULIN*	Innate immune response
		AllTRT	0.8					
17	52–54	cNurADG	8.7	−2.7	−0.61 (0.47)	−0.33 (0.06)	*NFATC2*	Immune response
		cNurMOR	2.7					

SSC, Sus scrofa chromosome; gVar, genetic variance; gCov, genetic covariance; rg, genetic correlation; qNurADG, average daily gain in the quarantine nursery; qNurHScore, health score in the quarantine nursery; cNurADG, average daily gain in the challenge nursery; cNurHScore, health score in the challenge nursery; cNurTRT, treatment rate in the challenge nursery; cNurMOR, mortality rate in the challenge nursery; FinADG, average daily gain in the finisher; FinHScore, health score in the finisher; FinTRT, treatment rate in the finisher; FinMOR, mortality rate in the finisher; AllTRT, treatment rate across the challenge nursery and finisher; AllMOR, mortality rate across the challenge nursery and finisher; CWT, carcass weight; DRESS, dressing percent; LYLD, lean yield; DBF, backfat; DLD, loin depth; ADFI, average daily feed intake; FIDuration, average daily feed intake duration; FInVisits, average daily number of feed intake visits; FIRT, average daily feed intake rate; ADWD, average daily water dispensed; ADWI, average daily water disappearance; WIDuration, average daily water intake duration; WInVisits, average daily number of water intake visits; WDRT, average daily water dispensed; WIRT, average daily water disappearance rate.

**Fig. 4.**
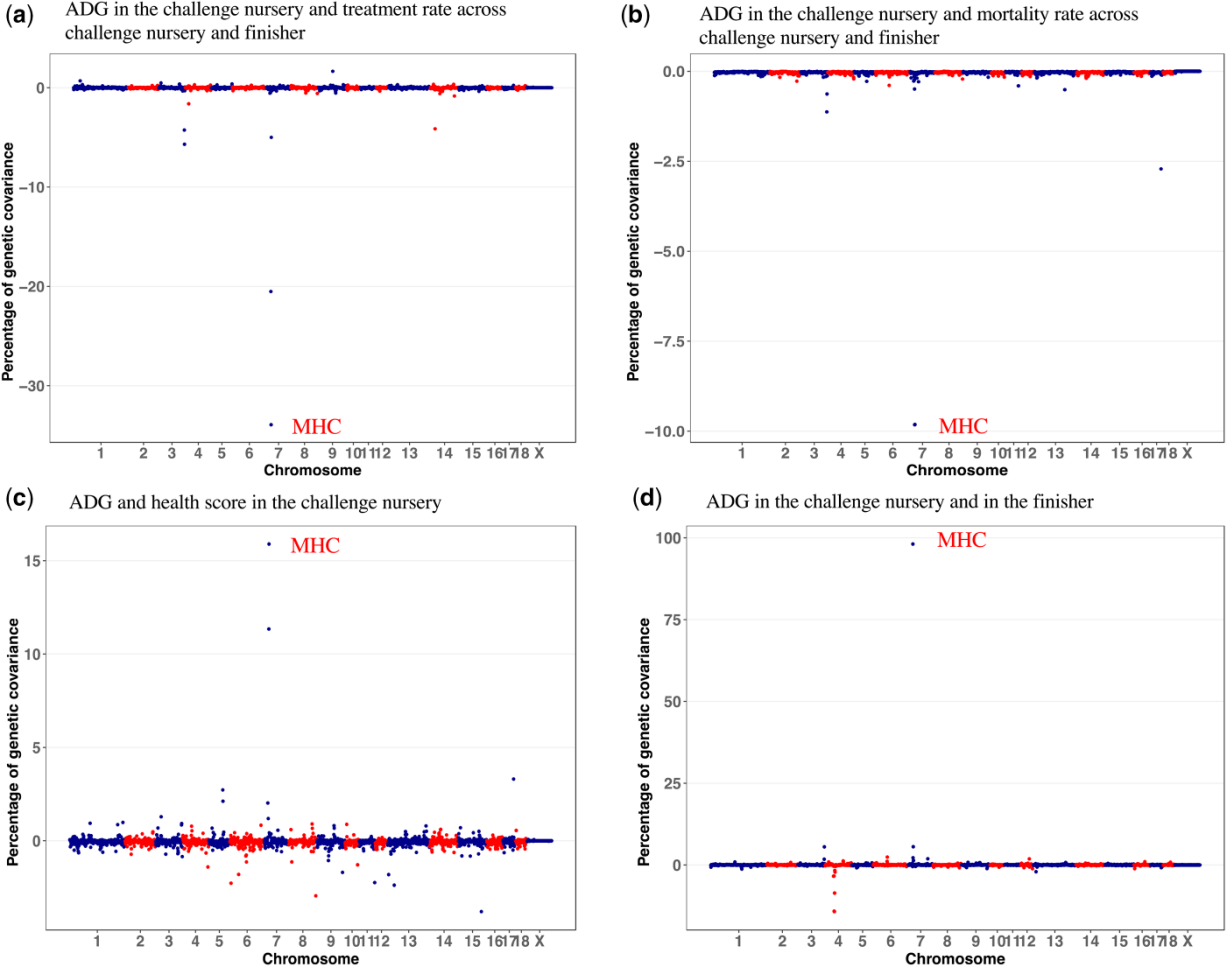
Percentage of genetic covariance explained by each nonoverlapping 1-Mb window across the genome for ADG in the challenge nursery with clinical disease traits and with ADG in the finisher based on bivariate Bayes-B analyses. MHC, major histocompatibility complex.

**Fig. 5.**
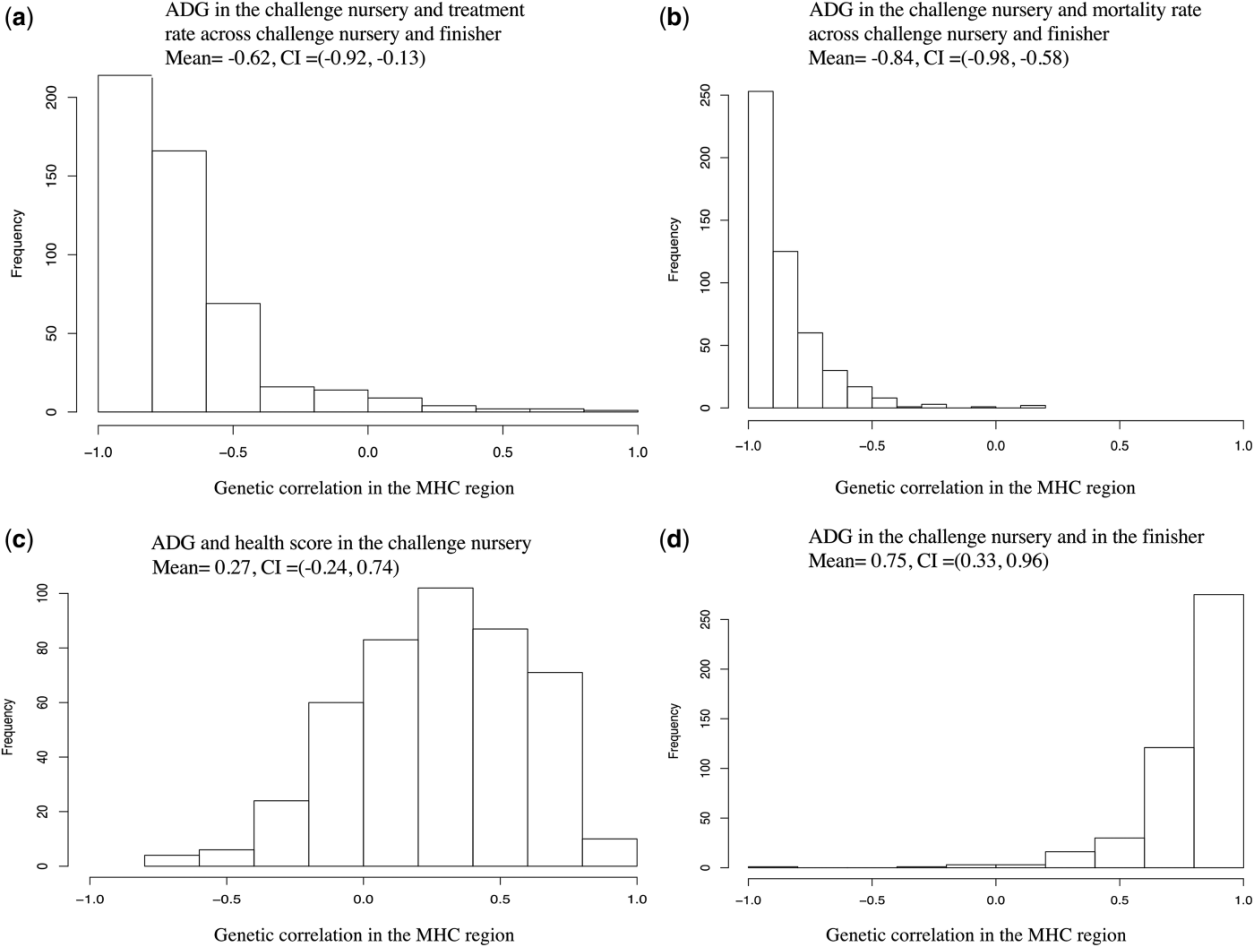
Posterior distribution of the genetic correlation in the MHC region for ADG in the challenge nursery with clinical disease traits and with ADG in the finisher based on bivariate Bayes-B analyses. Mean, posterior mean of the genetic correlation; CI, 95% credible interval.

Genetic correlations for the identified pleiotropic QTL regions, computed as the estimated genetic covariance explained by the 3-Mb window, divided by the square root of the product of the estimated genetic variances for the 3-Mb window for the 2 analyzed traits, were much stronger than the corresponding estimates of the genetic correlations across the genome. Genomic regions on SSC3 (127–130 Mb) and the MHC region (Fig. 4) were detected as pleiotropic QTL for many traits within and between production phases, including ADG, number of health treatments (TRT), mortality rate (MOR), and health score (HScore). Three pleiotropic QTL were detected for cNurADG with FinADG (Table 3), with genetic covariances between −37% and 107%, including the MHC region. cNurADG was estimated to be positively genetically correlated with FinADG for the QTL on SSC3 (127–130 Mb) and in the MHC region but negatively correlated for the QTL on SSC4. The posterior distribution of the genetic correlation between cNurADG and FinADG for the MHC region was skewed to the left, with a positive posterior mean of 0.75, which was much higher than for the whole genome, and a 95% confidence interval (CI) ranging from 0.33 to 0.96 (Fig. 5). Four pleiotropic QTL were identified for cNurADG with qNurADG, with estimated genetic covariance ratios ranging from −6.7% to 17.3%. It is noteworthy that the pleiotropic QTL on SSC7 (16–18 Mb) was close to the MHC region and was estimated to explain 17.3% of the genetic covariance.

Four pleiotropic QTL regions (genetic covariance estimates between −0.7% and −60%; Table 3) were detected for cNurADG, with TRT and with MOR, including in the MHC region, with moderate to high negative genetic correlation estimates (−0.43 to −0.85), and on SSC3 (127–130 Mb), also with moderate to high negative genetic correlation estimates (−0.43 to −0.75). All genetic correlation estimates for the MHC and for the SSC3 QTL region (127–130 MB) were much stronger than the corresponding whole genome genetic correlation estimates. Posterior distributions of the genetic correlations of ADG with clinical disease traits are shown in Fig. 4 and Supplementary Figs. 11 and 12. Posterior distributions of the genetic correlations of cNurADG with TRT and MOR for the MHC region (Fig. 5 and Supplementary Fig. 11) were skewed to the right, with the full 95% CI being below 0. Two pleiotropic QTL were identified for cNurADG with cNurHScore, including in the MHC region (27.4% of genetic covariance and genetic correlation equal to 0.27) and on SSC15 (−4.0% of genetic covariance and genetic correlation equal to −0.53). The posterior distribution of the genetic correlation between cNurADG and cNurHScore for the MHC region was slightly skewed to the left, with the 95% CI ranging from −0.24 to 0.74 (Fig. 5).

Four pleiotropic QTL were detected for qNurADG with HScore in the quarantine nursery (qNurHScore), with genetic covariance ratio estimates ranging from −2% to 4.6% (Table 3). Five pleiotropic QTL were identified for FinADG with traits other than cNurADG, i.e. with FinHScore, AllTRT, AllMOR, with genetic covariance ratio estimates ranging from −1.8% to 0.7%. A pleiotropic QTL for FinADG with AllTRT was also detected on SSC3 (127–130 Mb), with a moderate negative estimate of the genetic correlation (−0.4), but which was much stronger than the genetic correlation estimate for the whole genome.

### Fine-mapping of QTL in the MHC region

The MHC region was identified as the most important QTL region for many of the analyzed disease resilience traits. The MHC is a large and complex genomic region that includes many immunity-related genes (Hammer et al. 2020). Principle component analysis of genotypes across the whole genome showed a clear pattern of subpopulations but the MHC region did not, probably because the MHC region was not indirectly heavily selected for in the parental lines for the 7 sources of F1 pigs (Supplementary Fig. 13). SNP diversity was generally stable across the MHC region, ruling out the effect of allele frequencies (Supplementary Fig. 14).

**Fig. 6.**
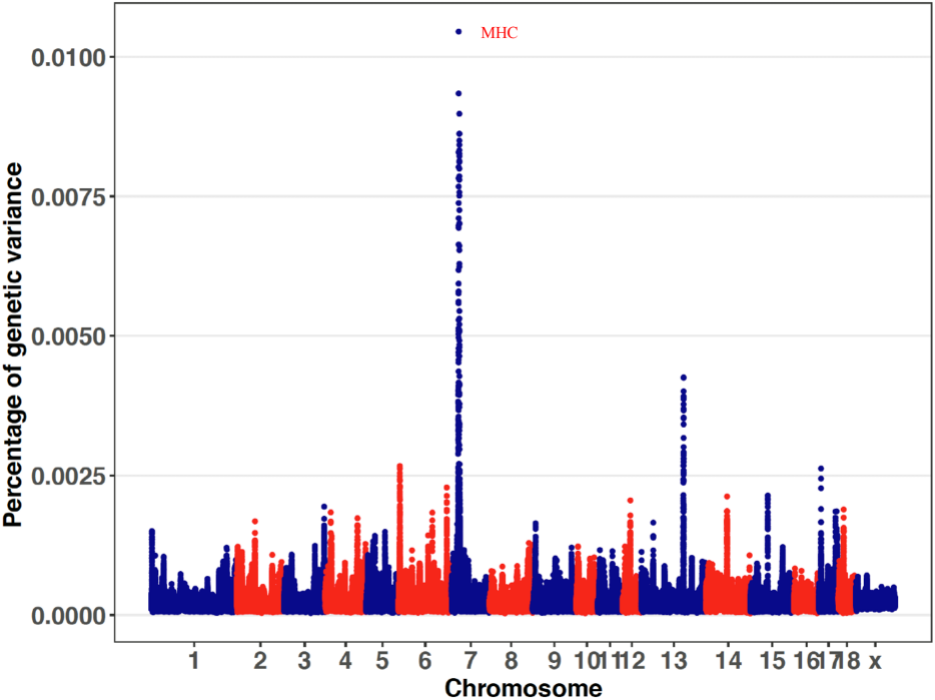
Percentage of genetic variance explained by putative QTL fitted each 5 kb across the genome for ADG in the challenge nursery based on the univariate Bayes-IM analysis. MHC, major histocompatibility complex.

**Fig. 7.**
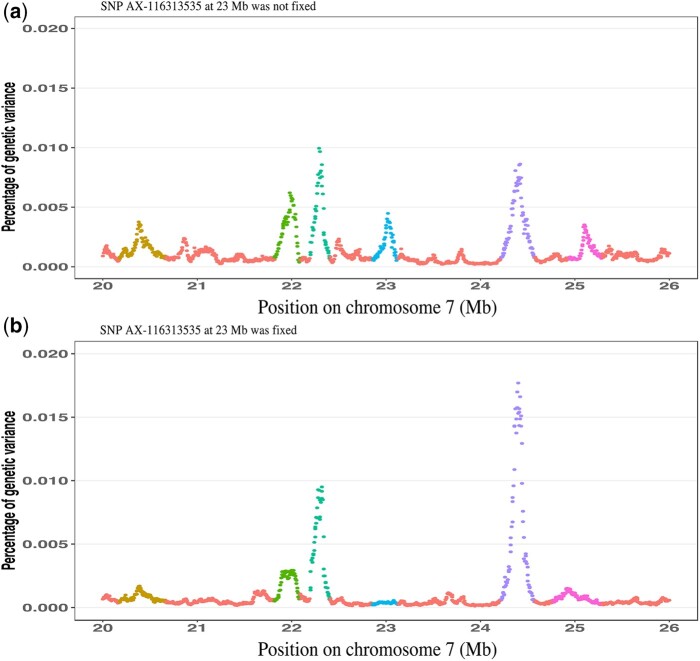
Fine-mapping results for the MHC region for ADG in the challenge nursery based on the univariate Bayes-IM analysis without a) or with b) genetic marker AX-116313535 fitted as a fixed covariate effect. Shown is the percentage of genetic variance explained by putative QTL fitted each 5 kb. MHC, major histocompatibility complex. Colors indicate the identified QTL regions based on a threshold of 0.1% of genetic variance. In (a), 6 QTL were identified at 20.175–20.67 Mb (QTL20), 21.815–22.095 Mb (QTL21), 22.19–22.405 Mb (QTL22), 22.85–23.13 Mb (QTL23), 24.215–24.575 Mb (QTL24), and 24.94–25.129 Mb (QTL25), which explained 0.15%, 0.16%, 0.17%, 0.10%, 0.30%, and 0.10% of genetic variance, respectively. In (b), the fixed genetic marker explained 7% of the genetic variance and captured all genetic variance at QTL23 and 3 additional QTL were identified, i.e. QTL21, QTL22, and QTL24, which explained 0.11%, 0.2%, and 0.4% of genetic variance, respectively.

**Fig. 8.**
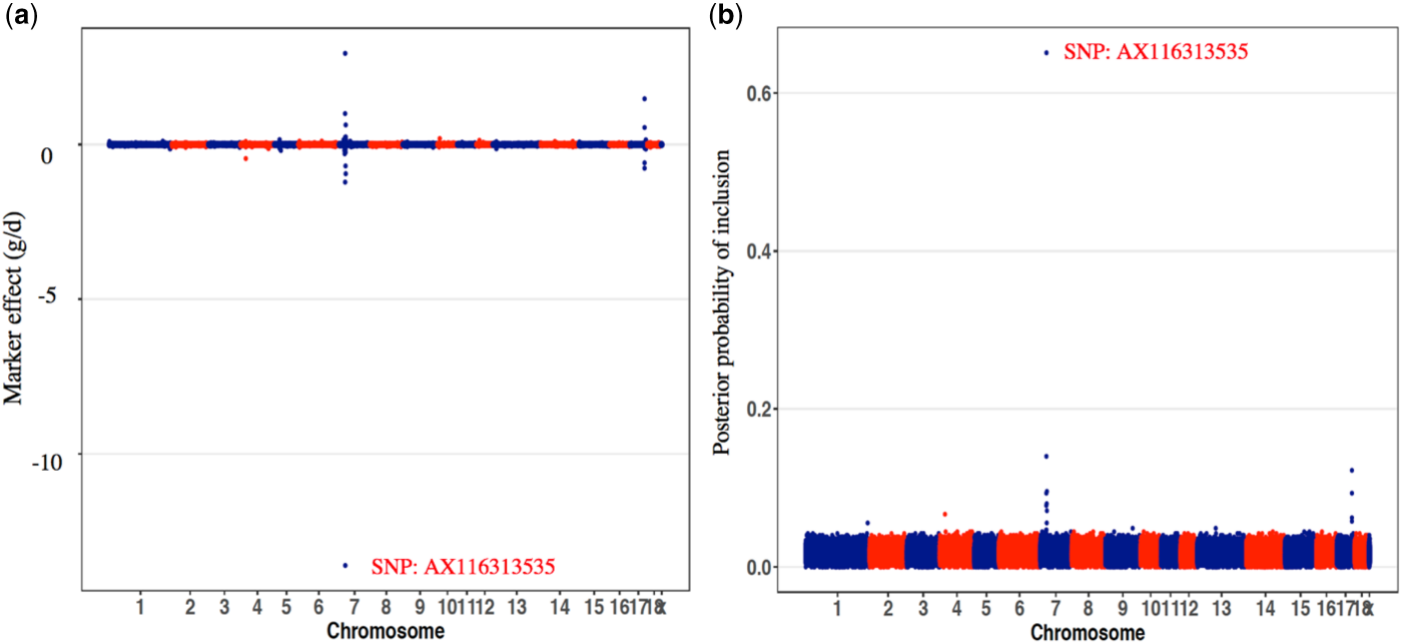
Estimates of marker allele substitution effects a) and posterior probabilities of inclusion b) for ADG in the challenge nursery based on the Bayes-B analysis. SNP AX116313535 is a genetic marker in the MHC and its negative allele substitution effect is for its major allele.

**Fig. 9.**
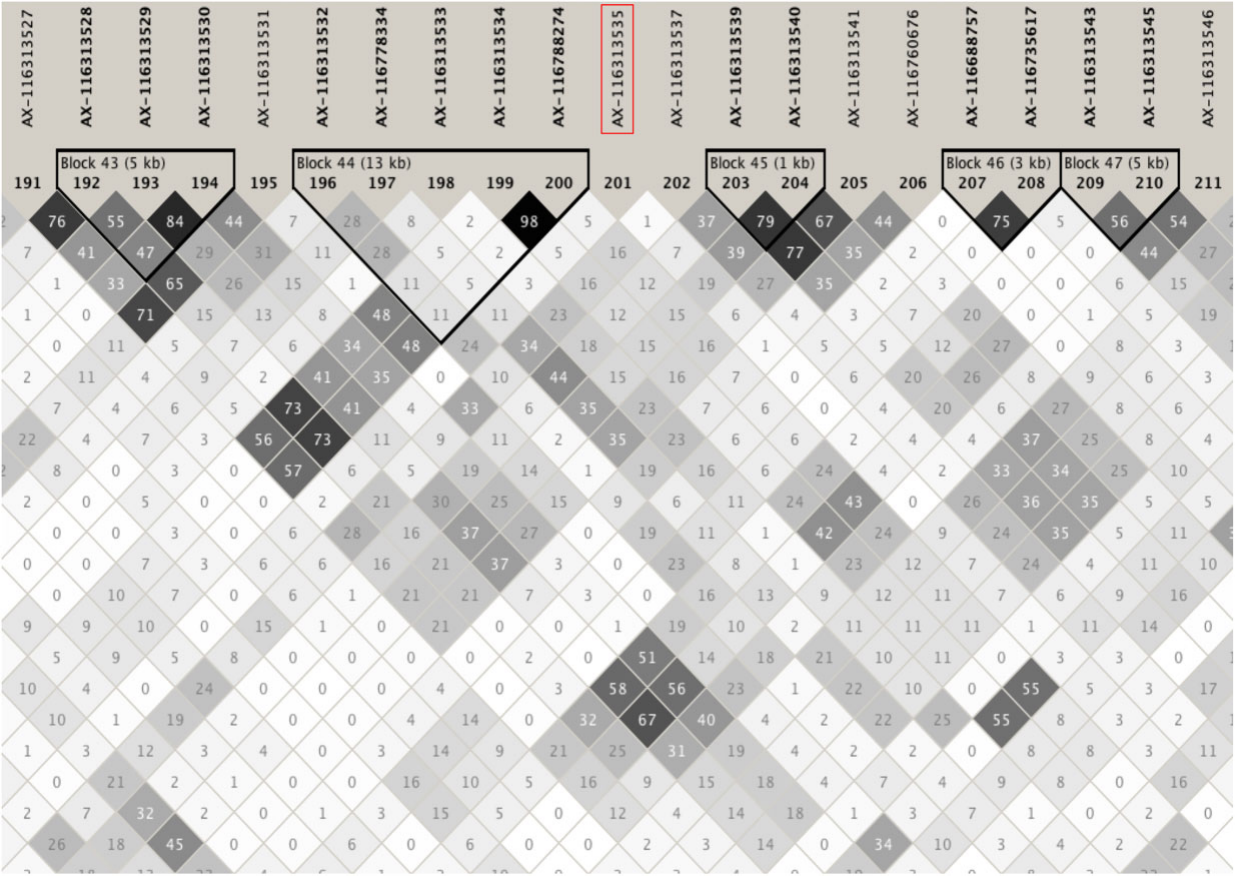
LD (r^2^) around genetic marker AX-116313535 in the MHC region based on the Haploview software (Barrett et al. 2005).

To further investigate the trait associations that were identified for the MHC, fine-mapping analyses were conducted for the whole genome (Fig. 6) and for the MHC region by fitting haplotype clusters rather than individual SNPs, using the Bayesian haplotype model Bayes-IM, developed by Kachman (2015). Figure 7 shows the results of the fine-mapping of QTL for cNurADG, with 6 QTL regions identified (Fig. 7a). However, the estimate of the genetic variance across these 6 QTL (1.6%) was much lower than that obtained in the univariate SNP-based Bayes-B GWAS (12.6%, Table 1) and even when using an SNP-based Bayes-C method (5.0%, results not shown). When investigating this discrepancy, it was noted that SNP AX-116313535 at 23,041,108 bp in the MHC region had a much larger allele substitution effect estimate for cNurADG in the SNP-based univariate Bayes-B analysis (−14 g/day or 0.24 genetic SD, explaining 3% of the genetic variance) than other SNPs in the region (−1 to 4 g/day) (Fig. 8a). This SNP also had a much higher probability of inclusion in the SNP-based Bayes-B analysis (0.65) than any other SNP in the MHC region (all <0.15) (Fig. 8b). Further analysis showed that this SNP was in low LD with other SNPs in the MHC region (Fig. 9), which resulted in the genotypes at this SNP not to be captured by the 8 haplotype clusters that were generated for this region in the Bayes-IM analysis, with frequencies of its major allele ranging from 0.5 to 0.98 across the 8 haplotype clusters. This explained the discrepancy in genetic variance captured by the MHC region based on the haplotype vs the SNP-based GWAS analyses. To address omission of the effect of this SNP in the subsequent Bayes-IM analyses, genotype at this SNP was fitted as an additional fixed covariate effect. The resulting estimate of the allele substitution effect (α) for cNurADG was −30 g/day, corresponding to 7% of the genetic variance (computed as 2pq$\alpha^{2}$, where *P* = 0.25 and *q* = 0.75, are the frequencies for alleles A and G, respectively, at this SNP, with genotypes coded as AA = 0, AG = 1, and GG = 2, and α is the estimate of the allele substitution effect). Results for the random SNP effects are in Fig. 7b. Compared with results from the original Bayes-IM analysis (Fig. 7a), 3 of the 6 originally identified QTL regions disappeared, in particular the region that includes the fixed SNP, as expected, which suggests that this SNP captured most of the genetic variance for these QTL. Based on these results, we declare presence of 4 putative QTL for the MHC region, in the 21, 22, 23 (captured by the SNP that was fitted as a fixed effect), and 24-Mb windows (Fig. 7b). In the following, these will be referred to as QTL21, QTL22, QTL23, and QTL24, respectively.

**Fig. 10.**
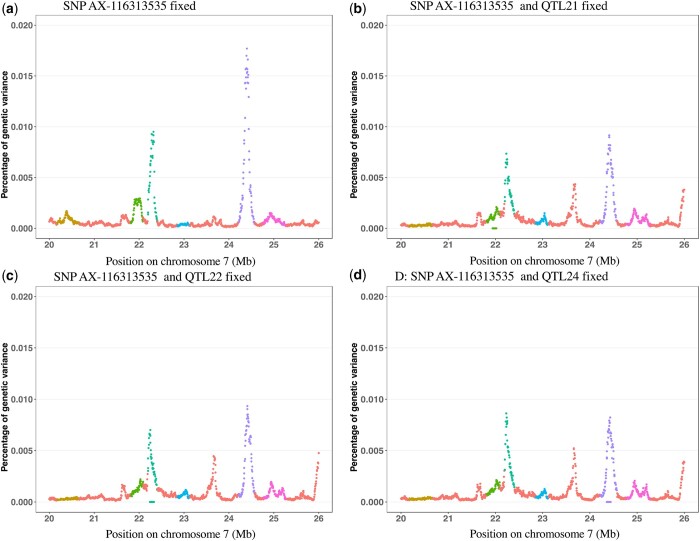
Fine-mapping results for the MHC region for ADG in the challenge nursery based on the univariate Bayes-IM analysis with different QTL and genetic marker AX-116313535 fitted as a fixed effect. Shown is the percentage of genetic variance explained by putative QTL fitted each 5 kb. Colors indicate the identified QTL regions based on a threshold of 0.1% of genetic variance.

To determine whether these 4 identified QTL regions are independent of each other or the result of LD between these regions, QTL21, 22, and 24 were also fitted as fixed effects in the Bayes-IM model, 1 at a time, along with the fixed effect of SNP AX-116313535 for QTL23, with results for the random SNP effects shown in Fig. 10. Note that the random components of the fitted model included all QTL, including those that were fitted as fixed. Results generally show that fitting a QTL as fixed reduced the proportion of genetic variance explained by the random effect for that QTL but did not completely eliminate it. However, fitting a QTL as fixed also reduced the genetic variance captured by other QTL, suggesting that these regions are not completely independent. For example, in Fig. 10c, fitting QTL22 as a fixed effect reduced the genetic variance for all other QTL, although they were still significant based on the 1% of genetic variance threshold. However, fitting QTL22 as fixed also led to the emergence of a new QTL in the 23-Mb window, which is likely because of the complex LD structure that is present in the MHC region.

**Fig. 11.**
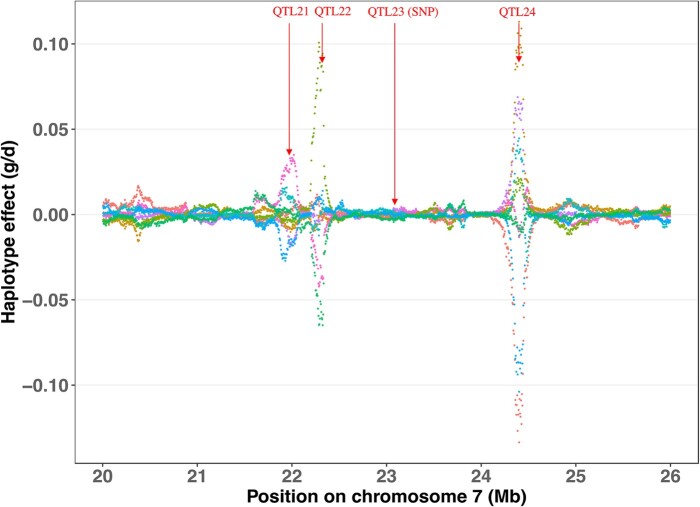
Estimates of the effects of the 8 haplotype clusters in the MHC region for ADG in the challenge nursery based on the Bayes-IM analysis. Vertical arrows indicate the position of the QTL identified in Fig. 7b. The SNP for QTL23 was fitted as a fixed effect and, therefore, does not show up in this figure. Each haplotype cluster is indicated by a different color.

Figure 11 shows estimates on cNurADG of the 8 haplotype clusters across the MHC region for the Bayes-IM model. The SNP AX-116313535 was fitted as a fixed effect, resulting in no effects of haplotypes in the QTL23 region. For the other 3 QTL, results showed that certain haplotypes may be more favorable than others for faster growth under the challenge.

**Fig. 12.**
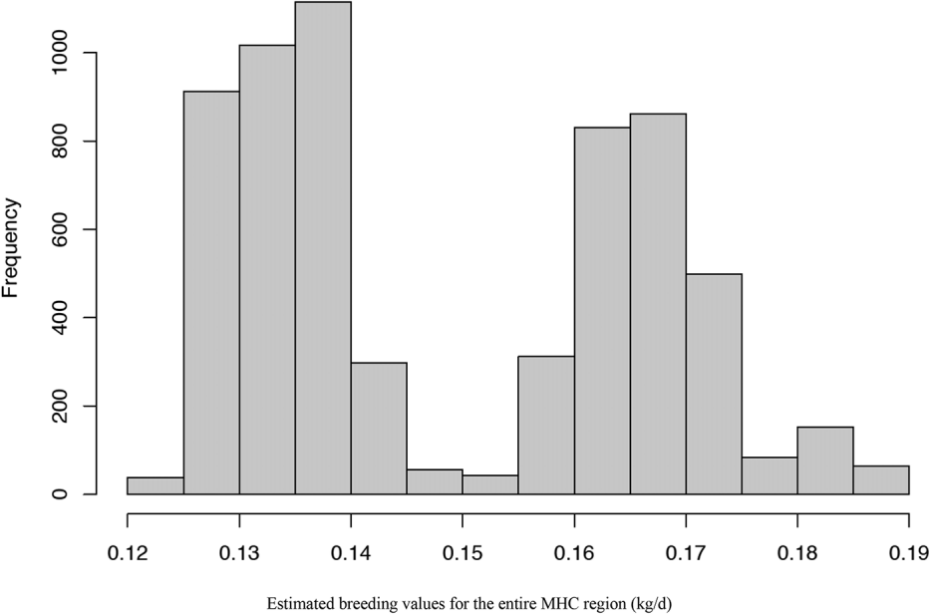
Distribution of EBVs for ADG in the challenge nursery for the expanded MHC region based on the Bayes-C analysis.

Distributions of estimated breeding value (EBV) for cNurADG for the entire MHC region and for each QTL are shown in Fig. 12 and Supplementary Fig. 15, respectively. The EBV were computed as the sum of the predictions of the random effects of haplotypes carried by an individual plus the fixed effect estimate of SNP AX-116313535. The EBV ranged from 0.12 to 0.19 kg/day, which represents a difference of 1.4 genetic SD units for cNurADG. It should be noted that most of the differences in EBV were caused by SNP AX-116313535.

The effects of SNP AX-116323535 were also estimated for each trait by fitting it as a fixed effect in the univariate genomic best linear unbiased prediction models of Cheng et al. (2020), using the ASReml software (Gilmour et al. 2015) (Table 2). Generally, the estimate of the genetic variance explained by this SNP was larger for growth rate and clinical disease traits under challenge (0.8–27.1%) than for carcass and feed and water intake traits (0–0.9%). It should be noted that these are based on fixed effect estimates of the SNP effects, which tend to be overestimated, as they are the sum of the true effect and the estimation error (Xu 2003). This resulted in the estimate of genetic variance explained by the SNP for some traits, e.g. FinTRT, to be even higher than the genetic variance explained by the entire MHC region based on the Bayes-B analyses. Among the traits with large effects of this SNP, ADG, and HScore had negative allele substitution effect estimates, while TRT and MOR had positive estimates, reflecting that homozygotes for the minor allele (AA) had higher ADG and HScore and lower TRT and MOR.

Fine-mapping analyses in the MHC region using Bayes-IM were also conducted for other traits for which the MHC region was identified as a QTL region in the SNP-based GWAS (Table 1). In all these analyses, SNP AX-116313535 was fitted as a fixed covariate effect. Results are shown in Supplementary Fig. 16. In these analyses, multiple peaks were identified but none reached the 1% of genetic variance threshold. A peak near QTL21 that was detected for cNurADG was also evident for WInVisits, a peak on QTL22 was evident for AllMOR and WIDuration, a peak near QTL23 was evident for all 4 analyzed traits, i.e. AllTRT, AllMOR, WIDuration, and WInVisits, and a peak at QTL24 was evident for WIDuration and WInVisits. However, the % of genetic variance captured by these peaks was much smaller than for the QTL detected for cNurADG, as the MHC region explained a much lower % of genetic variance for these 4 traits (Table 2).

### Gene set enrichment analyses

Although multiple “significant” QTL were identified in the GWAS, its statistical power remains limited, resulting in many false negatives. To evaluate the relevance and characterize the full spectrum of the GWAS results, beyond just the detected QTL, GSEAs were performed using the GSEA software (Subramanian et al. 2005), separately for each trait. The GSEA analyses used 3 customized libraries for enrichment; 1 derived from the QTL database for pigs (Hu et al. 2007), 1 based on the porcine transcriptome database of differentially expressed (DE) genes related to infection and health (Van Renne et al. 2018), and 1 based on the GO database (Subramanian et al. 2005). Each library was created by assigning their respective features to each nonoverlapping 0.25-Mb window of the genome. The % genetic variance associated with each 0.25-Mb window based on the univariate GWAS was used as weight in the GSEA for each trait. Results for each library are described in the following. The *q*-value threshold used to identify features that were “significantly” enriched was chosen separately for each of the 3 libraries in order to include an informative number of features in the presentation of results, without compromising readability.

#### QTL library enrichment analyses

**Fig. 13.**
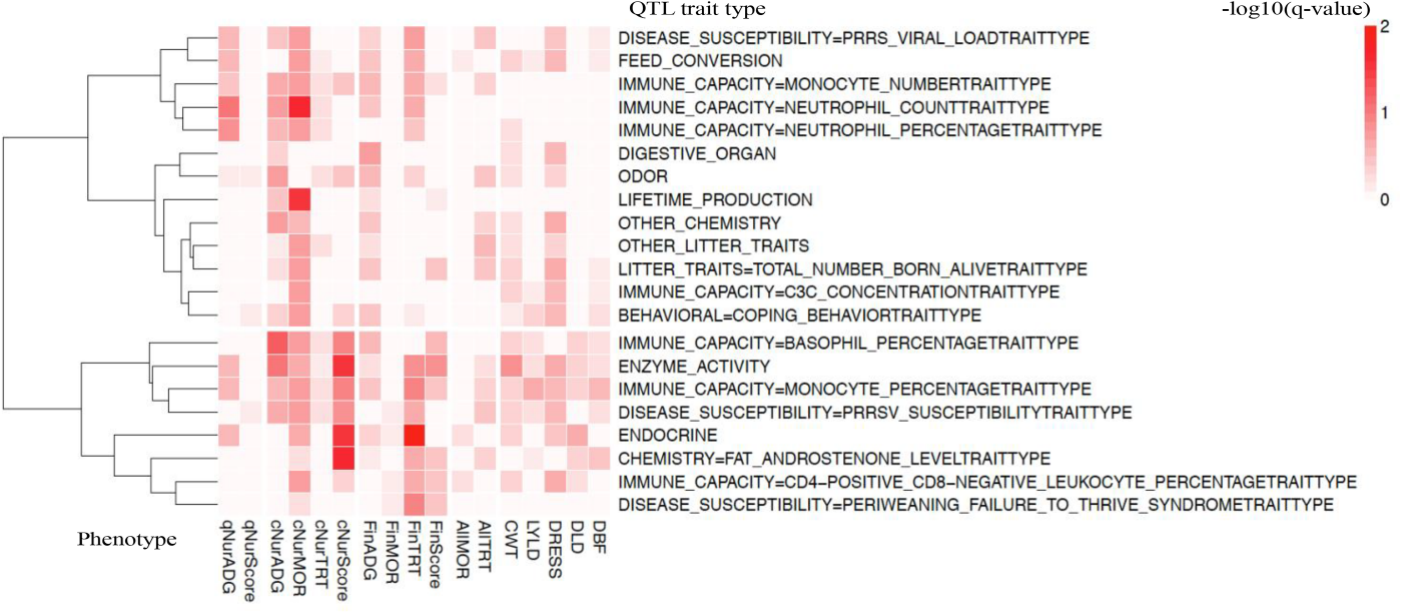
Heatmap of gene set enrichment analysis of GWAS results from univariate Bayes-B analyses using the QTL database library, showing the −log 10 of the *q*-value for enrichment of QTL trait types that had a *q*-value <0.2 for at least 1 analyzed trait. QTL, quantitative trait locus; qNurADG, average daily gain in the quarantine nursery; qNurHScore, health score in the quarantine nursery; cNurADG, average daily gain in the challenge nursery; cNurHScore, health score in the challenge nursery; cNurTRT, treatment rate in the challenge nursery; cNurMOR, mortality rate in the challenge nursery; FinADG, average daily gain in the finisher; FinHScore, health score in the finisher; FinTRT, treatment rate in the finisher; FinMOR, mortality rate in the finisher; AllTRT, treatment rate across the challenge nursery and finisher; AllMOR, mortality rate across the challenge nursery and finisher; CWT, carcass weight; DRESS, dressing percent; LYLD, lean yield; DBF, backfat; DLD, loin depth.

**Fig. 14.**
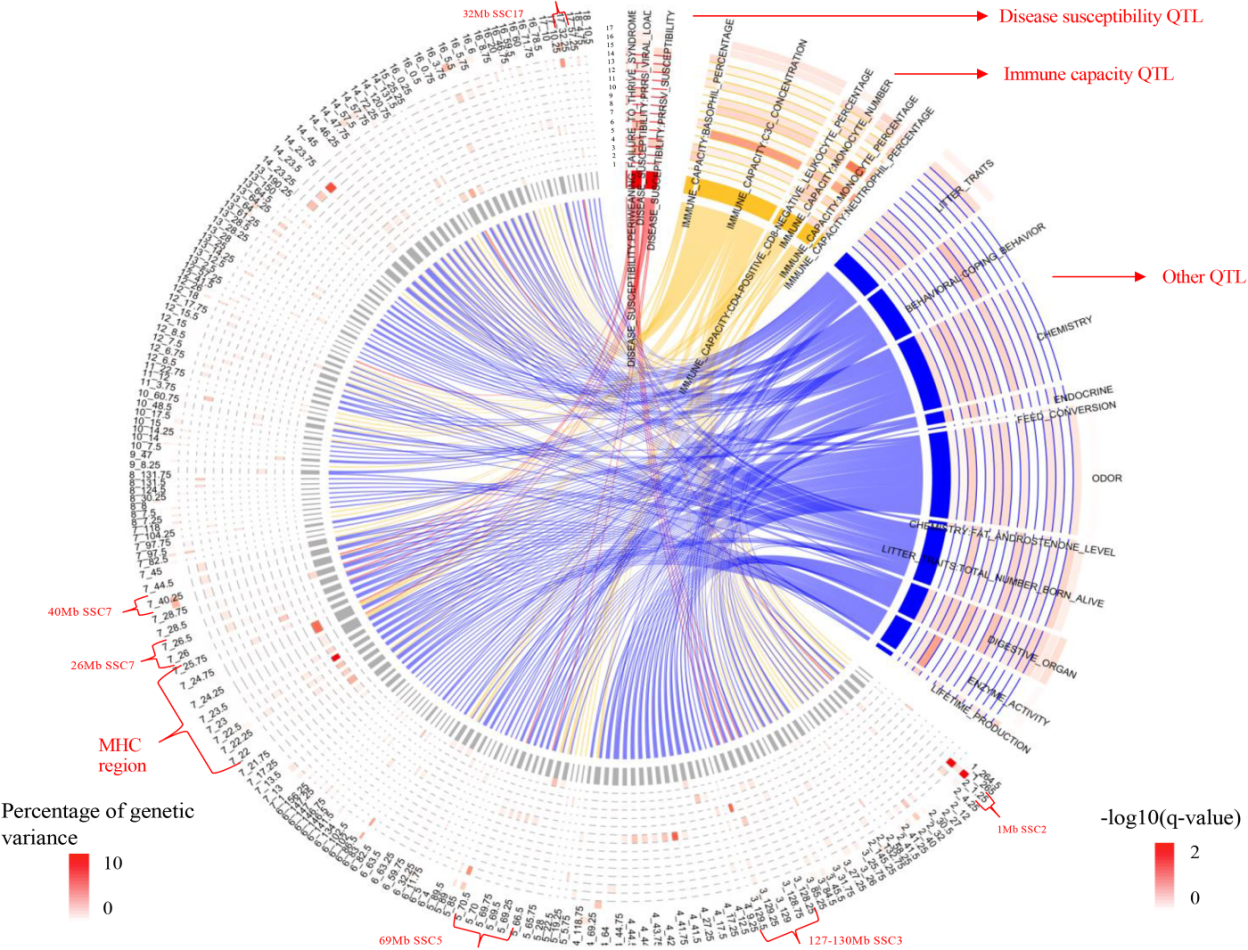
Overlap on the genome between significantly enriched QTL trait types and 0.25-Mb genomic windows that explained more than 0.2% of genetic variance in the univariate Bayes-B GWAS. MHC, major histocompatibility complex. 1:17 indicates trait name, which are 1: qNurADG, average daily gain in the quarantine nursery; 2: qNurHScore, health score in the quarantine nursery; 3: cNurADG, average daily gain in the challenge nursery; 4: cNurMOR, mortality rate in the challenge nursery; 5: cNurTRT, treatment rate in the challenge nursery; 6: cNurHScore, health score in the challenge nursery; 7: FinADG, average daily gain in the finisher; 8: FinMOR, mortality rate in the finisher; 9: FinTRT, treatment rate in the finisher; 10: FinHScore, health score in the finisher; 11: AllMOR, mortality rate across the challenge nursery and finisher; 12: AllTRT, treatment rate across the challenge nursery and finisher; 13: CWT, carcass weight; 14: LYLD, lean yield; 15: DRESS, dressing percent; 16: DLD, loin depth; 17: DBF, backfat.

The heatmap of GSEA results for QTL from the QTL database is shown in Fig. 13. Only QTL trait types that were enriched among 0.25-Mb windows for at least 1 analyzed trait at a *q*-value <0.2 are shown. The most enriched QTL were those for neutrophil count, lifetime production, enzyme activity, endocrine, and fat androsterone level. Enriched immune capacity QTL included QTL for monocyte number and percentage, neutrophil count and percentage, C3C concentration, basophil percentage, and CD4-positive and CD8-negative-related leukocyte percentage. Enriched disease susceptibility QTL included QTL for PRRS viral load, PRRS susceptibility, and periweaning failure-to-thrive syndrome. Figure 14 shows the overlap on the genome between the significantly enriched QTL types and 0.25-Mb genomic windows that explained more than 0.2% of genetic variance in the univariate Bayes-B GWAS. In Fig. 14, the QTL types were clustered into 3 groups, i.e. disease susceptibility, immune capacity, and others. The identified GWAS QTL in the MHC region (22–25 Mb on SSC7) overlapped with both disease susceptibility and immune capacity QTL, especially the 24-Mb region, as did the 40-Mb QTL region on SSC7. The 128–129-Mb QTL region on SSC3, the 69-Mb QTL region on SSC5, the 26-Mb QTL region on SSC7, and the 32-Mb QTL region on SSC17 only coincided with disease susceptibility QTL, while the 1-Mb QTL region on SSC2 only coincided with immune capacity QTL.

#### Porcine transcriptome enrichment analysis

**Fig. 15.**
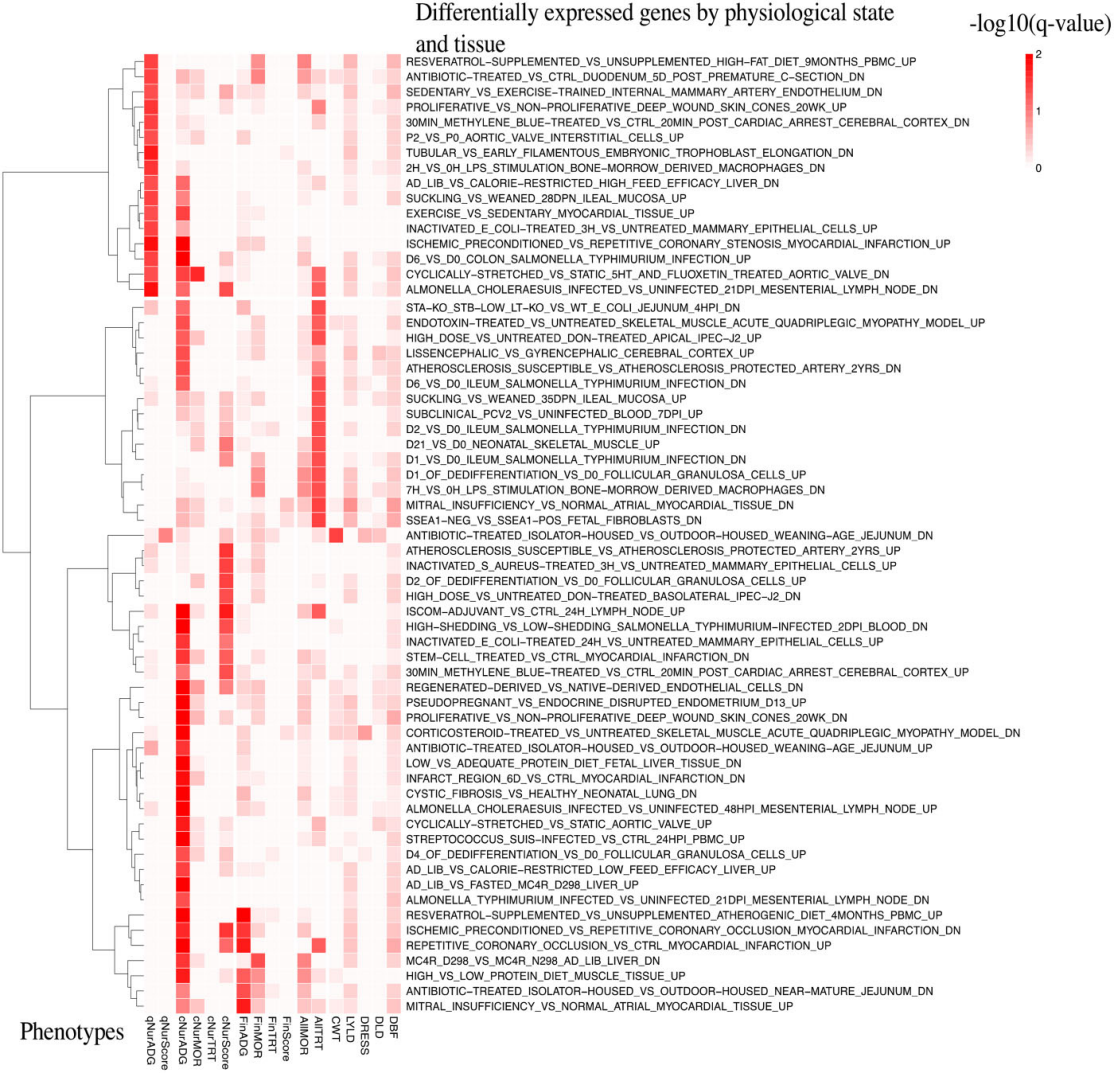
Heatmap of gene set enrichment analysis of GWAS results from univariate Bayes-B analyses using the porcine transcriptome database library, showing the −log 10 of the *q*-value for enrichment of differentially expressed genes that had a *q*-value <0.05 for at least 1 analyzed trait. qNurADG, average daily gain in the quarantine nursery; qNurHScore, health score in the quarantine nursery; cNurADG, average daily gain in the challenge nursery; cNurHScore, health score in the challenge nursery; cNurTRT, treatment rate in the challenge nursery; cNurMOR, mortality rate in the challenge nursery; FinADG, average daily gain in the finisher; FinHScore, health score in the finisher; FinTRT, treatment rate in the finisher; FinMOR, mortality rate in the finisher; AllTRT, treatment rate across the challenge nursery and finisher; AllMOR, mortality rate across the challenge nursery and finisher; CWT, carcass weight; DRESS, dressing percent; LYLD: lean yield; DBF, backfat; DLD, loin depth.

The heatmap of GSEA results for DE genes from the porcine transcriptome database is shown in Fig. 15. Only DE genes that were enriched at a *q*-value <0.05 among 0.25-Mb windows for at least 1 of the analyzed traits are shown. Genomic regions associated with qNurADG were most enriched for genes that were down-regulated in bone marrow derived macrophages at 2 h following lipopolysaccharide (LPS) stimulation and in the mesenterial lymph node at 21 days post *Salmonella choleraesuis* infection, and for genes that were up-regulated 3 h following treatment of mammary epithelial cells with inactivated *Escherichia coli* and 6 days after *Salmonella typhimurium* infection in colon. Genomic regions associated with cNurADG and cNurHScore in the challenge nursery and with AllTRT across the challenge nursery and finisher were enriched for genes that were down-regulated in mesenterial lymph node at 21 days after *S. choleraesuis* infection, in the ileum at 1, 2, and 6 days following infection with *S. typhimurium*, in blood at 2 days after high shedding *S. typhimurium* infection, in the jejunum at 4 h after DestA *E. coli* infection, and in bone morrow derived macrophages at 7 h following LPS stimulation. These genomic regions were also enriched for genes that were upregulated in mesenterial lymph node at 48 h after *S. choleraesuis* infection, in the colon at day 6 after *S. typhimurium* infection, in mammary epithelial cells at 3 and 24 h after inactivated *E.coli* treatment, in peripheral blood mononuclear cells at 24 h after *S. suis* infection, in blood at 7 days after subclinical PCV2 infection, and in the adjuvant lymph node at 24 h following immune-stimulating complexes (ISCOM) stimulation.

**Fig. 16.**
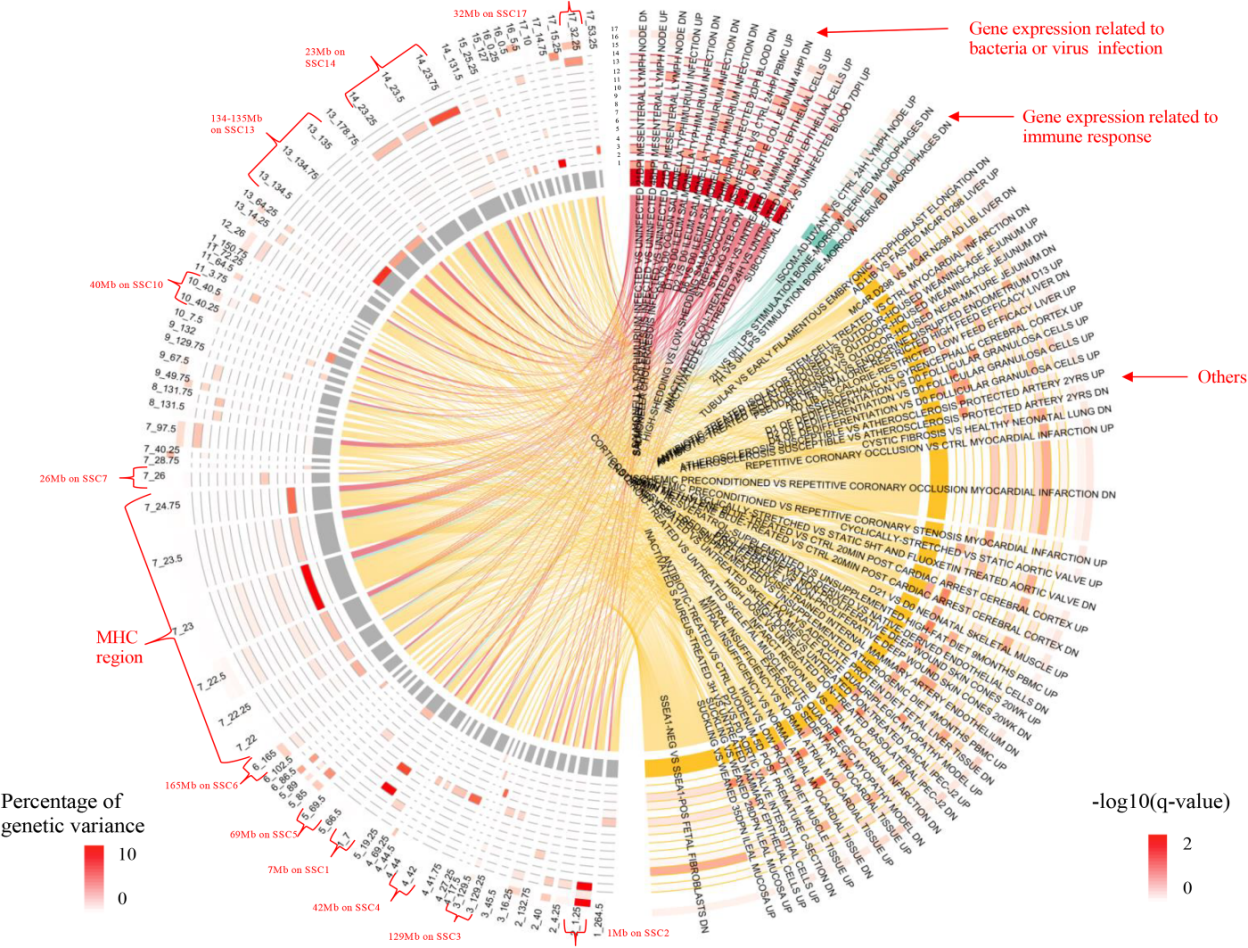
Overlap on the genome between the significantly enriched differentially expressed genes and 0.25-Mb genomic windows that explained more than 0.5% of genetic variance in the univariate Bayes-B GWAS. MHC, major histocompatibility complex. 1:17 indicates trait name, which are 1: qNurADG, average daily gain in the quarantine nursery; 2: qNurHScore, health score in the quarantine nursery; 3: cNurADG, average daily gain in the challenge nursery; 4: cNurMOR, mortality rate in the challenge nursery; 5: cNurTRT, treatment rate in the challenge nursery; 6: cNurHScore, health score in the challenge nursery; 7: FinADG, average daily gain in the finisher; 8: FinMOR, mortality rate in the finisher; 9: FinTRT, treatment rate in the finisher; 10: FinHScore, health score in the finisher; 11: AllMOR, mortality rate across the challenge nursery and finisher; 12: AllTRT, treatment rate across the challenge nursery and finisher; 13: CWT, carcass weight; 14: LYLD, lean yield; 15: DRESS, dressing percent; 16: DLD, loin depth; 17: DBF, backfat.

Figure 16 shows the overlap on the genome between significantly enriched DE genes and 0.25-Mb genomic windows that explained more than 0.5% of genetic variance in the GWAS for at least 1 of the analyzed traits. The DE genes were categorized based on response to bacterial and viral infection, immune stimulation, and others. The MHC region (22–24 Mb on SSC7) overlapped with the largest number of DE genes, in particular with DE genes related to bacterial and viral infection. There was also a small portion of the MHC that overlapped with DE genes in response to stimulation by LPS and ISCOM. Several other QTL regions on SSC13 (134–135 Mb) and SSC14 (23 Mb) also overlapped with many DE genes, in particular DE genes in response to bacterial and viral infection, while a small portion of the QTL region on SSC14 (23 Mb) overlapped with DE genes in response to immune stimulation. Several other QTL regions on SSC1, 2, 3, 4, 5, 6, 7, 10, and 17 also overlapped with DE genes in response to bacterial and viral infection.

#### GO enrichment analysis

**Fig. 17.**
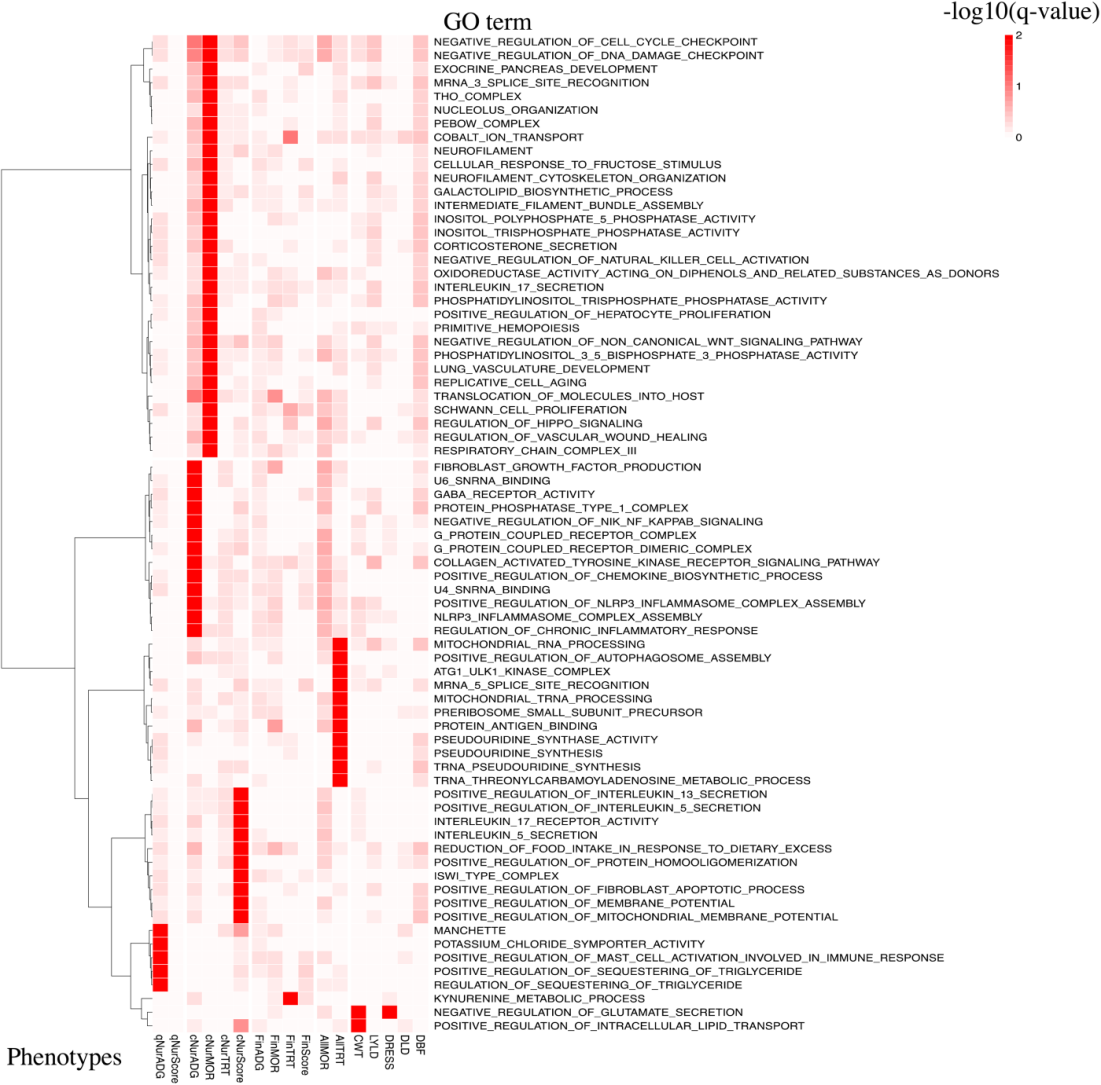
Heatmap of gene set enrichment analysis of GWAS results from univariate Bayes-B analyses using the GO library, showing the −log 10 of the *q*-value for enrichment of GO terms that had a *q*-value <0.01 for at least 1 analyzed trait. qNurADG, average daily gain in the quarantine nursery; qNurHScore, health score in the quarantine nursery; cNurADG, average daily gain in the challenge nursery; cNurHScore, health score in the challenge nursery; cNurTRT, treatment rate in the challenge nursery; cNurMOR, mortality rate in the challenge nursery; FinADG, average daily gain in the finisher; FinHScore, health score in the finisher; FinTRT, treatment rate in the finisher; FinMOR, mortality rate in the finisher; AllTRT, treatment rate across the challenge nursery and finisher; AllMOR, mortality rate across the challenge nursery and finisher; CWT, carcass weight; LYLD, lean yield; DRESS, dressing percent; DLD, loin depth; DBF, backfat.

The heatmap of GSEA results based on the GO library is shown in Fig. 17. Only GO terms that were enriched at a *q*-value <0.01 among 0.25-Mb windows for at least 1 analyzed trait are shown. Genomic regions associated with qNurADG were enriched for genes that play a role in positive regulation of mast cell activation involved in immune response. Genomic regions associated with cNurADG were enriched for genes involved in NLRP3 inflammasome complex assembly and regulation of chronic inflammatory response. Genomic regions associated with cNurMOR were enriched for genes involved in interleukin 17 secretion and negative regulation of natural killer cell activation. Genomic regions associated with cNurHScore were enriched for genes involved in interleukin activity.

**Fig. 18.**
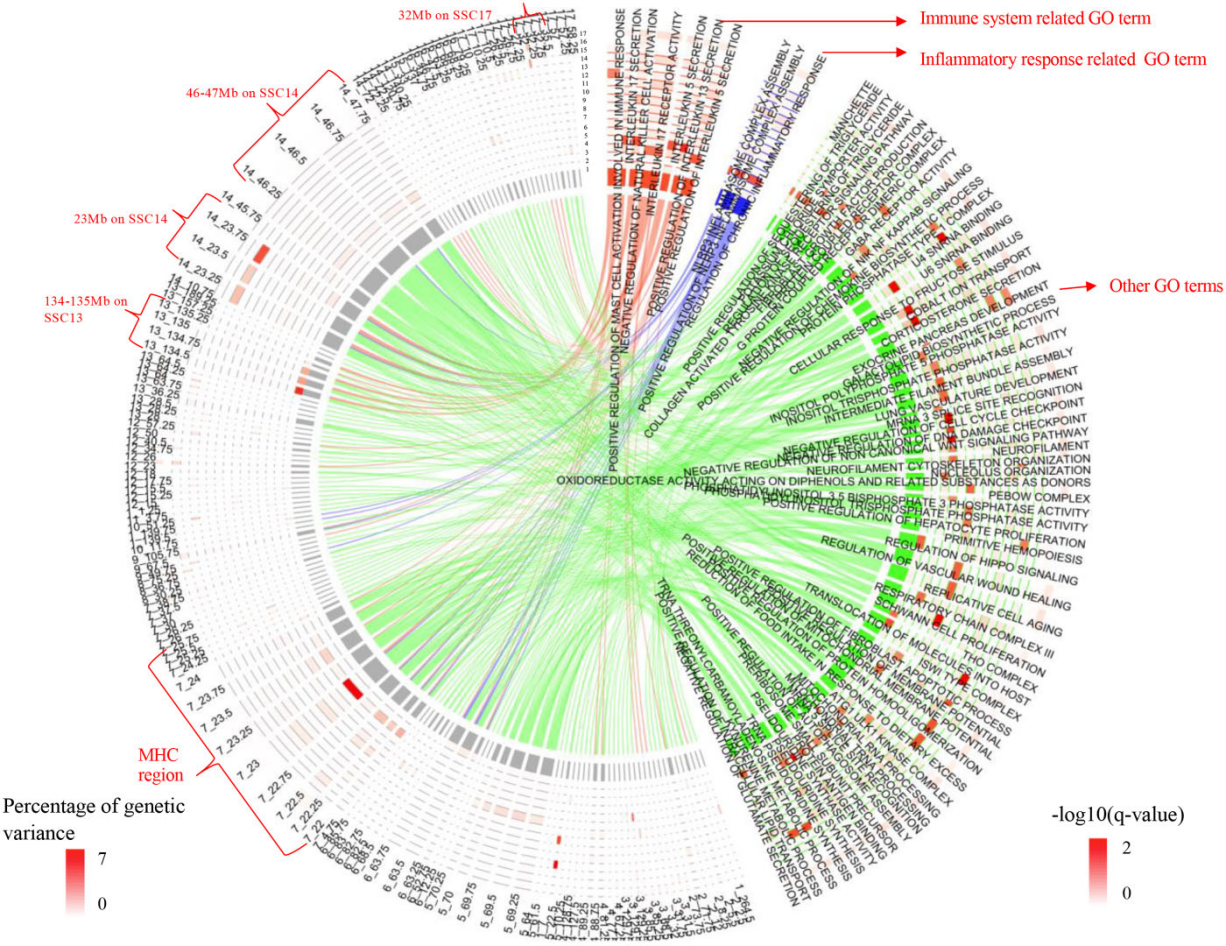
Overlap on the genome between the significantly enriched GO terms and 0.25-Mb genomic windows that explained more than 0.1% of genetic variance in the univariate Bayes-B GWAS. MHC, major histocompatibility complex. 1:17 indicates trait name, which are 1: qNurADG, average daily gain in the quarantine nursery; 2: qNurHScore, health score in the quarantine nursery; 3: cNurADG, average daily gain in the challenge nursery; 4: cNurMOR, mortality rate in the challenge nursery; 5: cNurTRT, treatment rate in the challenge nursery; 6: cNurHScore, health score in the challenge nursery; 7: FinADG, average daily gain in the finisher; 8: FinMOR, mortality rate in the finisher; 9: FinTRT, treatment rate in the finisher; 10: FinHScore, health score in the finisher; 11: AllMOR, mortality rate across the challenge nursery and finisher; 12: AllTRT, treatment rate across the challenge nursery and finisher; 13: CWT, carcass weight; 14: LYLD, lean yield; 15: DRESS, dressing percent; 16: DLD, loin depth; 17: DBF, backfat

Figure 18 shows the overlap on the genome of the significantly enriched GO terms and 0.25-Mb genomic windows that explained more than 0.1% of the genetic variance. The significant GO terms were clustered into 3 groups, i.e. immune system, inflammatory response, and others. The MHC region on SSC7 (22–24 Mb) and other QTL regions on SSC13 (134–135 Mb) and SSC14 (23 and 46–47 Mb) overlapped with multiple significant GO terms, of which the MHC region and the 23-Mb region on SSC14 overlapped with significant GO terms for both immune and inflammatory response, while the QTL regions on SSC13 (134–135 Mb) and SSC17 (32 Mb) only overlapped with the immune response GO term, and the QTL region on SSC14 (46–47 Mb) only with the inflammatory response GO term.

## Discussion

To our knowledge, this is the first GWAS for a broad spectrum of performance and clinical disease traits that were collected in pigs under disease, including growth and carcass performance, clinical disease traits, and feeding and drinking behavior traits. Data were from the novel natural polymicrobial disease challenge model described by Putz et al. (2019) and Cheng et al. (2020). A large number of pigs (3,285 pigs) with genotypes from a high-density SNP chip panel (650,000 SNPs) were used for the GWAS, which is larger than most previous GWAS studies in pigs. The MHC region was consistently identified for multiple traits, including the class I, II, and III subregions. In the following, the most important results will be discussed, starting with results from the GSEAs, followed by results on specific QTL and the MHC.

### Gene set enrichment analyses

Genomic regions that were found to be associated with traits recorded in the natural disease challenge model were enriched for features from the 3 libraries that were used for the GSEA, i.e. for previously reported disease susceptibility and immune capacity QTL, for genes that were differentially expressed following bacterial or viral infection and immune response, and for GO terms related to immune and inflammatory response. The natural disease challenge included various bacterial and viral diseases, including *Salmonella*, *S. suis*, PRRSV, porcine rotavirus A, B and C, and influenza A, and pigs were vaccinated for PCV2, starting with batch 7. These results suggest that the genomic regions identified in this study are important for the host to defend against these and other pathogens.

The immune capacity QTL, DE genes, and GO terms that were found to be enriched in the GWAS results are well connected to leukocytes, which play an important role in the immune system. Previously published immune capacity QTL that were enriched in the GWAS results were mainly identified for leukocyte level (Fig. 14), including basophils, neutrophils, monocyte, and lymphocytes, which are cells of the immune system that are involved in protecting the body against infectious diseases. Previously published DE genes related to immune response that were enriched in the GWAS results were genes that were involved in bone marrow derived macrophages following LPS stimulation and in the adjuvant lymph node following ISCOM stimulation (Fig. 16). These DE genes were also related to leukocytes, specifically monocytes and lymphocytes. Monocytes are the largest type of leukocyte and can differentiate into macrophages and influence the process of adaptive immunity. Lymphocytes include different types of white blood cells of the immune system, including natural killer cells, B cells, and T cells, which are the main types of cells found in lymph nodes. GO terms related to the immune system that were enriched among the GWAS results were primarily the terms of interleukins, natural killer cells, and mast cells (Fig. 18). Interleukins are a group of cytokines expressed by leukocytes and largely determine the function of the immune system. A natural killer cell is a type of cytotoxic lymphocyte that is critical to the innate immune system. Mast cells are another white blood cell that are very similar to basophils in appearance and function. Enriched GO terms related to inflammatory response were mainly the terms of *NLRP3* inflammasome and chronic inflammatory response. *NLRP3* is expressed predominantly in macrophages, which are involved in the innate immune system. Chronic inflammation is predominated by macrophages, lymphocytes, and plasma B cells.

The enriched QTL and DE genes related to disease susceptibility or bacterial and viral infection also agreed well with the pathogens that were present in the disease challenge model. Enriched disease susceptibility QTL were mainly related to response to PRRSV infection, which is a virus that was ubiquitous in the disease challenge model. Enriched DE genes related to bacterial or viral infection were mainly in response to infections by *S. suis*, *E. coli*, PCV, and especially *Salmonella*, of which, *S. suis* and *Salmonella* were identified to be present in the disease challenge model, while pigs that entered the disease challenge model were vaccinated for PCV2, starting with batch 7.

### Candidate genes in the identified QTL regions

Multiple QTL regions identified in the GWAS overlapped with enriched features from the 3 libraries, i.e. disease susceptibility and immune capacity QTL, bacterial, or viral infection and immune response-related DE genes, and immune system and inflammatory response-related GO terms (Figs. 13, 15, and 17). Candidate genes located in these regions will be discussed in the following.

The 32-Mb QTL region on SSC17 overlapped with enriched features from all 3 libraries (Figs. 13, 15, and 17) and harbors the gene *MAVS*. This gene encodes an intermediary protein in the virus-triggered beta interferon signaling pathways that is required for activation of transcription factors that regulate expression of beta interferon and is essential to drive antiviral innate immunity in response to infection by RNA viruses (Kumar et al. 2006), such as PRRSV and rotavirus, which were present in the disease challenge model. The importance of this region suggests that part of the immune response was triggered by RNA virus infection. This QTL region was identified for LYLD and DBF (1.3–2.3% of genetic variance; Table 1), indicating that RNA virus infection may affect carcass composition.

The QTL region at 1 Mb on SSC2 was also identified for LYLD and DBF (explaining 6.6–10.5% of the genetic variance) and overlapped with enriched immune capacity QTL and DE genes related to bacterial or viral infection. This QTL region harbored several genes, i.e. *IGF2*, *IFITM1*, *CD81*, and *LSP1*. The *IGF2* gene has previously been shown to be important for muscle mass and fat deposition of pigs (Nezer et al. 1999), while *IFITM1*, *CD81*, and *LSP1* are immune related genes and could affect carcass traits through the disease challenge. The *IFITM1* gene encodes interferon-induced transmembrane protein 1, which is involved in immune response signaling and has been identified as an antiviral restriction factor for influenza A virus replication (Feeley et al. 2011); influenza was also diagnosed in the disease challenge model. The *LSP1* gene encodes lymphocyte-specific protein 1, which is expressed in lymphocytes, neutrophils, macrophages, and endothelium, and can regulate neutrophil motility (Wang et al. 2002). The *CD81* protein associates with *CD4* and *CD8*, which play a role in T-cell recognition and activation by binding to their respective class II and class I MHC ligands on an antigen presenting cell and provides a costimulatory signal with *CD3* on T cells (Levy et al. 1998).

The 127–130-Mb QTL region on SSC3, the 69-Mb QTL region on SSC5, and the 26-Mb QTL region on SSC7 overlapped with both enriched disease susceptibility QTL and DE genes related to bacterial or viral infection; the 26-Mb QTL region on SSC7 overlapped with both bacterial or viral infection and with immune response-related DE genes (Figs. 13 and 15). The 127–130-Mb QTL region on SSC3 harbors the *RSAD2* gene, which encodes a virus inhibitory protein called viperin that inhibits many DNA and RNA viruses, including influenza (Mattijssen and Pruijn 2012). This QTL region was associated with multiple traits, i.e. cNurTRT, FinADG, DBF, and ADFI (explaining 1–2.7% of the genetic variance; Table 1). Bivariate GWAS also found that this QTL region contributed a negative genetic correlation for ADG with MOR or TRT (−0.40 to −0.75; Table 3) and a positive genetic correlation for cNurADG with FinADG (0.41). Thus, viral infection in the disease challenge may activate *RSAD2* expression, which decreases growth rate and increases treatment and mortality rates. Lim et al. (2021) also found that the expression of *RSAD2* in the quarantine nursery had sizable correlations with resilience traits, using expression data on a subset of the animals used here.

The 69-Mb QTL region on SSC5 for cNurHScore (1.4% of genetic variance) harbors the *IL17RA* gene, which is related to defense against several bacterial and fungal infections (Lévy et al. 2016). The 26-Mb QTL region on SSC7 was also detected for cNurHScore (1.2% of genetic variance) and harbors the *TINAG* gene, which encodes a basement membrane glycoprotein that was initially identified as a target of antibodies in some forms of immunologically mediated tubulointerstitial nephritis (Katz et al. 1992). Therefore, these 2 genes might be associated with clinical phenotypes such as low health scores when pigs are under a disease challenge.

The 134–135-Mb QTL region on SSC13 overlapped with enriched DE genes related to bacterial or viral infection and with GO terms related to immune system (Figs. 15 and 17). This QTL region harbors the *NRROS* gene, which is related to host defense against bacterial and fungal infections (Noubade et al. 2014). This QTL region also explained 7% of the genetic variance for qNurADG (Table 1) and contributed a positive genetic correlation of qNurADG with cNurADG (0.55; Table 3). Although qNurADG was collected in the quarantine nursery, which was free of major pathogens, pigs may have been exposed to some other minor pathogens, likely bacteria or fungi, which was also evident from the gene expression studies by Lim et al. (2021), possibly resulting in an association of this gene with qNurADG. The positive genetic correlation between qNurADG and cNurADG in this QTL region indicates that this gene may also have an effect in the challenge nursery.

The 23-Mb QTL region on SS14 overlapped with enriched DE genes that were related to both bacterial or viral infection and immune response and with enriched GO terms that were related to both immune system and inflammatory response. This QTL region harbors the gene *ULK1*, which encodes *unc-51* like autophagy activating kinase 1, which plays a central role in interferon-dependent immunity (Saleiro et al. 2016). This QTL region also explained 5.1% of the genetic variance for AllTRT, suggesting it is associated with clinical disease.

Some other QTL regions overlapped with only 1 enriched feature but also harbored immune related genes (Figs. 15 and 17). The 7-Mb QTL region on SSC1 and the 40-Mb QTL region on SSC10 only overlapped with enriched DE genes for bacterial or viral infection but harbors 2 immune related genes, *IGF2R* and *TAGAP*. Interestingly, *IGF2R* encodes a receptor for *IGF2*, which is located in the QTL region at 1 Mb on SSC2, which was detected for the carcass traits LYLD and DBF. The *TAGAP* gene codes for a GTPase-activating protein that plays important roles during T-cell activation (Connelly et al. 2014). This QTL region explained 2.1–4.2% of the genetic variance for mortality. Variation in the *TAGAP* gene may contribute to the genetic variation observed for mortality but *IGF2R* may also play a role. In addition to its association with carcass traits, *IGF2* has also been shown to have a stimulating effect on T-lymphocyte proliferation, which is mediated by the binding of *IGF2* to its receptor (Brown et al. 1985). The *IGF2* gene also plays key roles in mitogenic activity, antiapoptosis, and in immune system regulation (Kim et al. 2006).

The 40-Mb QTL region on SSC10 harbors the gene *MAP3K8*, which promotes the production of *TNF-alpha* and *IL-2* during T-lymphocyte activation and plays a critical role in innate immunity (Mielke et al. 2009). This QTL region explained 2% of genetic variance for qNurADG and contributed a positive genetic correlation of qNurADG with cNurADG (0.58). This suggests that this gene may be activated in both the quarantine and challenge nursery.

The 46–47-Mb QTL region on SSC14 only overlapped with enriched GO terms for inflammatory response but harbors the gene *LIF*, which has a pivotal role in T-cell immunity (Metcalfe 2011). This QTL was not detected in the univariate GWAS but was identified in the bivariate GWAS, with a strong negative genetic correlation between cNurADG and cNurMOR (−0.74). This suggests that *LIF* may affect both cNurADG and cNurMOR but in different directions, as expected.

Using the same data as used here, Jeon et al. (2021) showed that the SNP WUR000125 at 127 Mb on SSC4 is significantly associated with cNurADG, cNurTRT, and AllTRT. This SNP was previously associated with host response to experimental infection of nursery pigs with PRRSV (Boddicker et al. 2012) and is in high LD with the putative causative mutation for this effect in the *GBP5* gene (Koltes et al. 2015), which plays a role in immune regulation and in mediating inflammatory immune response in the mouse (Shenoy et al. 2012). Our univariate GWAS results showed that the QTL region (126–128 Mb on SSC4) that harbors this SNP explained 0.17-0.28% of genetic varianction for cNurADG, cNurTRT, and AllTRT. Although relatively low, this is higher than most other regions, confirming the importance of this SNP.

### Role of MHC in disease resilience

The MHC class I and II subregions contain the MHC class I and class II series of genes of the adaptive immune system that are related to peptide presentation. The MHC class III subregion does not include MHC genes but harbors other key immunity-related genes that are important for immune defense mechanisms and inflammation (Hammer et al. 2020).

#### QTL detected in the MHC region

The MHC region explained from 1.3% to 12.8% of the genetic variance for different traits (Table 1), with the largest effect for cNurADG. The univariate GWAS identified different parts of the MHC region to be associated with different traits, e.g. the class I subregion for FinADG, ADFI, and FIRT, the class II subregion for WIDuration and WInVisis, and all 3 subregions for cNurADG (Table 1). These differences could be due to the fact that the different MHC subregions harbor genes with different functions but could also reflect false negatives or positives. The MHC region also contributed a strong negative genetic correlation of cNurADG with TRT or MOR (−0.62 to −0.85; Table 3) and a positive genetic correlation with FinADG (0.79), suggesting the MHC genes are pleiotropic for these traits, in the expected directions.

The fine-mapping analyses identified 4 QTL in the MHC region for cNurADG (Fig. 7). QTL21 and QTL22 were located outside the MHC region, toward the 3′ end, QTL23 was located in the class I subregion, and QTL24 was mostly in the class III subregion but also included the centromere and part of the class II subregion (Supplementary Fig. 17). Although not declared significant, a low peak was present in the class II subregion. Although these 4 QTL were not found to be completely independent of each other, multiple QTL related to disease resilience are likely present in the MHC region.

The SNP AX-116313535 in the QTL23 region (MHC class I subregion) was found to have a large effect for many disease resilience traits. This SNP is located inside a weak enhancer region close to the *TRIM39* gene (Herrera-Uribe et al. 2020), which is involved in innate immune response (Suzuki et al. 2016). This gene was significantly overexpressed 6 h after LPS treatment (Herrera-Uribe et al. 2020). Herrera-Uribe et al. (2020) identified a signal of this enhancer in porcine alveolar macrophages 6 h following LPS and Poly(I:C) stimulation but this region also showed a low signal when under control or 2 h following LPS and Poly(I:C) stimulation. This suggests that the enhancer that harbors this SNP may regulate the expression of the *TRIM39* gene and, thereby, affect immune response. This SNP was not identified in previous studies that identified large effects of the MHC region on IgG level and viral load in blood of pigs following infection with PRRSV and PCV (Serão et al. 2014; Walker et al. 2018; Sanglard et al. 2020) because it was not included in the SNP panels that they used (Porcine SNP60 and GGP Porcine HD chip). This SNP was in low LD with other SNPs in the MHC and its effects may, therefore, not have been captured in these previous GWAS.

The MHC region overlapped with enriched features identified in the GSEA for all 3 libraries (Figs. 13, 15, and 17). Specifically, the MHC class III and class II subregions overlapped with the disease susceptibility and immune capacity QTL, the MHC class I and part of the class III subregion overlapped with enriched DE genes for both bacterial or viral infection and immune response, while the class II subregion overlapped with enriched DE genes for only bacterial or viral infection. For enriched GO terms, the MHC class I subregion overlapped with enriched terms for both immune and inflammatory response, but identified parts of the class I and the class III subregions only overlapped with enriched GO terms for immune response. Different parts of the MHC region overlapped with different features but all class I, II, and III subregions overlapped with enriched features from at least 1 of the 3 libraries.

### Comparison of MHC results with other livestock species

The QTL harbored in the MHC region for pig, chicken, and cattle were compared using the Animal QTL database (QTLdb) developed by Hu et al. (2007). In the pig MHC region, in total, 120 QTL associated with health, production, reproduction, and meat and carcass traits were identified previously, mostly associated with health-related traits (69 QTL), followed by meat and carcass related traits (47 QTL). Additionally, Rothschild et al. (1986), Renard and Vaiman (1989), and Matsubara et al. (2018) also found that MHC haplotypes were associated with pig growth and reproduction. The MHC region is much smaller in chickens (about 0–200 kb on chromosome 16, Fulton et al. 2016) than in pigs, with 71 QTL identified in previous studies. About half (37) of these QTL were related to health, followed by production traits (27 QTL). For cattle, 123 QTL have been identified in the MHC region (27–28 Mb on chromosome 23; Ellis and Hammond 2014), however, most of these were associated with milk production (62 QTL), followed by health-related traits (35 QTL). Our GWAS results, coupled with these previous findings, demonstrate that the MHC region is important for livestock, not only for health but also for production efficiency, which may be because animals have to spend extra energy on immune response when under challenge, which is an energetically demanding process (Ganeshan et al. 2019).

Our results also demonstrate the importance of the *TRIM39* gene for disease resilience, as the enhancer for this gene harbored in the MHC Class I subregion harbored an SNP with large effects. The *TRIM39* gene is conserved in many species, including cattle, chicken, and sheep (Jaratlerdsiri et al. 2014). The amino acid sequences of the *TRIM39* protein are also highly conserved in mammals (Pan et al. 2011), indicating the importance of this gene in other species.

### Selection for disease resilience

Previously, Mallard et al. (1998) selected pigs for 8 generations for high, low, and controlled immune responsiveness, using EBV for antibody and cell-mediated immune responses, and found that the high line had significantly higher antibody responses to various antigens. Moreover, after infection with *Mycoplasma hyorhinis*, the high line pigs had significantly less peritonitis and pleuritis and a higher rate of gain compared with the low and control lines. In chickens, Pinard et al. (1992) reported on a selection experiment based on total antibody titer in blood plasma 5 days after immunization of 37-day-old chickens with sheep red blood cells. The results of 9 generations of divergent selection suggested that high or low immunocompetence can be selected for. The 2 lines (high and low) differed in the frequencies of certain MHC haplotypes, which were responsible for a significant proportion of the within-line variation in antibody production (Pinard et al. 1993). Lamont et al. (2003) applied index selection for multiple immune traits in chickens, including antibody response, cellular response, and phagocytic activity measured 3 weeks after vaccination, and also found significant changes in MHC haplotype frequencies in the selected lines. Selection for improved immune resistance is complex, as selection for an improved adaptive immune response against a particular pathogen may compromise immune response against a different pathogen (Kaufman, 2018). However, there is potential to select for increased innate immune responses, potentially leading to increased robustness or resistance to a wide spectrum of pathogens. Using data from the natural polymicrobial disease challenge model that was employed here, Putz et al. (2019) and Cheng et al. (2020) found that resilience to multiple pathogens is heritable and, thus, can be selected for. It is, however, possible that the responses observed in this disease challenge and its associated GWAS were dominated by PRRSV infection. Thus, further study is necessary to ensure that selection on the QTL regions identified here confers resilience to multiple pathogens.

Our GWAS results in the MHC also show that certain haplotypes were more favorable than others for faster growth rate (more resilient) under a polymicrobial disease challenge. One explanation for the observed haplotype effects could be that multiple QTL with independent effects are required to maintain the functions of coordinated sets of alleles (Trowsdale and Knight 2013) and particularly the SNP AX-116313535, which had the largest effects. Therefore, these favorable haplotypes can potentially be used to select for disease resilience under a multiple disease challenge.

The strong negative genetic correlations of cNurADG with TRT/MOR that were estimated for the MHC region and the positive genetic correlations of cNurADG with cNurHScore suggest that pigs with the favorable alleles or haplotypes for the MHC region grew faster under the disease challenge, had fewer medical treatments, were less likely to die, and had a higher health score. The SNP AX-116313535 in the class I subregion was estimated to explain 0.8–27.1% of the genetic variance for ADG, TRT, MOR, and HScore (Table 2), which also makes it possible to select for this SNP alone to increase disease resilience. Note that favorable genotype AA at this SNP had a low frequency (5%), which implies substantial potential to select for the A allele to increase disease resilience.

## Conclusions

The MHC and other QTL that harbor immune related genes were identified to be associated with disease resilience traits in a large-scale natural polymicrobial disease challenge. Genomic regions associated with disease resilience were enriched for previously published QTL related to disease susceptibility and immune capacity, for functional pathways related to inflammatory and immune response, and for genes that were differentially expressed following bacteria or virus infection and immune stimulation. The MHC region was pleiotropic for growth rate in the challenge nursery and in the finisher and for treatment and mortality rates. Growth rate in the challenge nursery showed strong negative genetic correlations in the MHC region with treatment and mortality rates (−0.62 to −0.85) and a strong positive genetic correlation with growth rate in the finisher (0.79). Four QTL were identified in the MHC region, with 1 SNP in the class I subregion capturing the largest effects, explaining 0.8–27.1% of the genetic variance for growth rate and multiple clinical disease traits under the disease challenge. The favorable allele at this SNP had a frequency of 25%, indicating substantial potential for genetic improvement. In summary, the MHC and other QTL that were identified play an important role in host response to infectious diseases and can be incorporated in selection to improve disease resilience.

## Ethical approval

The animal experiments were carried out in accordance with the recommendations of the Canadian Council on Animal Care (Putz et al. 2019; Cheng et al. 2020) and the protocol approved by the Animal Care and Use Committee at the University of Alberta (AUP00002227).

## Data availability

The data analyzed in this study were collected on animals that were provided by and are part of the commercial breeding programs of the 7 investing member businesses of PigGen Canada (https://piggencanada.org/, last accessed 12/30/2021). As such, the data and samples generated on these animals are confidential and protected as intellectual property or as trade secrets. As a result, the data analyzed in this study are not publicly available but are stored in a secure data base at the University of Alberta. Data can, however, be made available on reasonable request, as detailed in supplementary file “Data access procedure.”

Supplemental material is available at *G3* online.

## Supplementary Material

jkab441_Supplemental_Figures_and_Tables

jkab441Supplemental_Material
