# Supplementary material for: Genome-wide association study of disease resilience traits from a natural polymicrobial disease challenge model in pigs identifies the importance of the major histocompatibility complex region
Source: G3 (Bethesda). 2021 Dec 28;12(3):jkab441. doi: 10.1093/g3journal/jkab441 (PMC9210302; doi:10.1093/g3journal/jkab441)
Supplement: jkab441_Supplemental_Figures_and_Tables [file jkab441_supplemental_figures_and_tables.pdf]

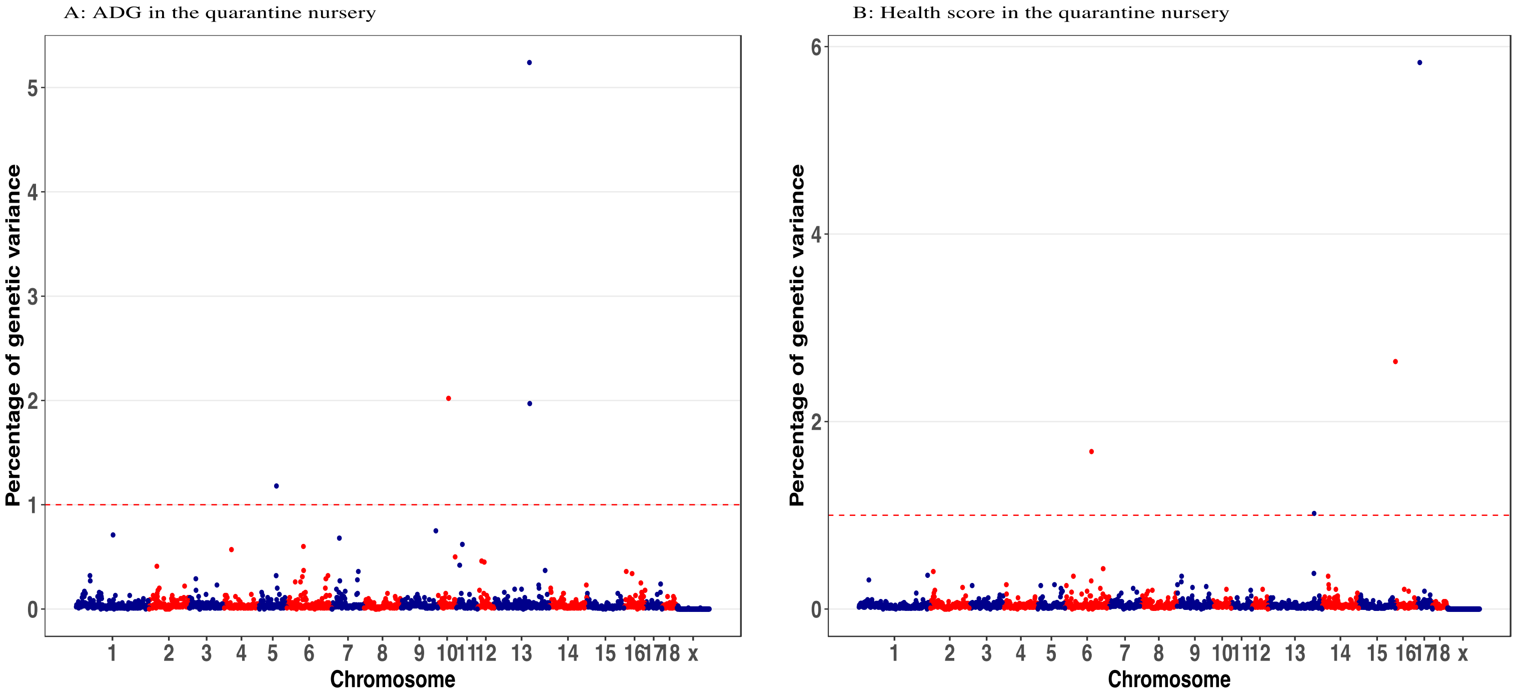

Figure S1. Percentage of genetic variance explained by each non-overlapping 1 Mb window across the genome for average daily gain (ADG) and health score in the quarantine nursery based on univariate GWAS Each dot indicates 1-Mb genomic window and univariate GWAS was performed using BayesB with Pi = 0.999.

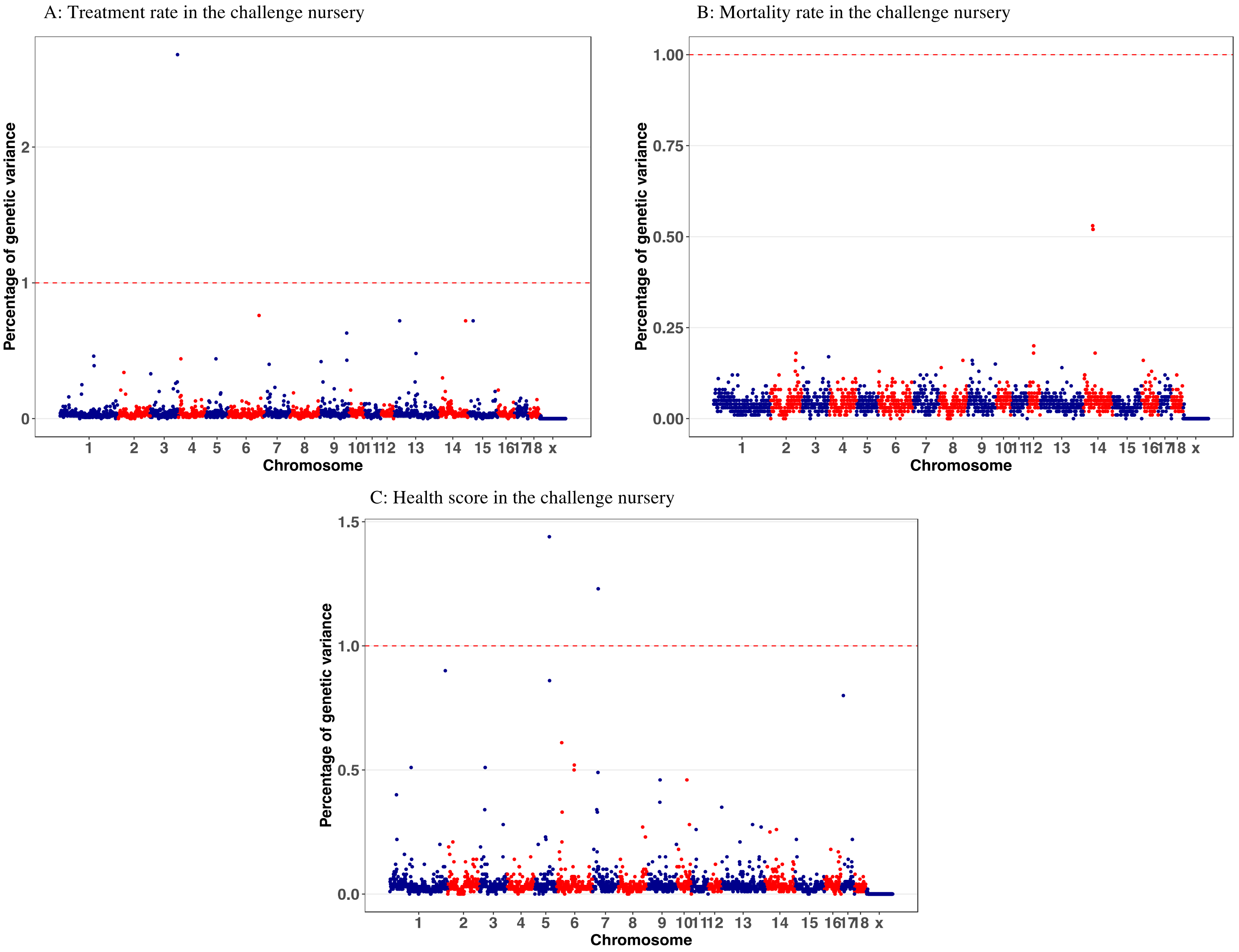

Figure S2. Percentage of genetic variance explained by each non-overlapping 1 Mb window across the genome for treatment rate, mortality rate, and health score in the challenge nursery based on univariate GWAS Each dot indicates 1-Mb genomic window and univariate GWAS was performed using BayesB with Pi = 0.999.

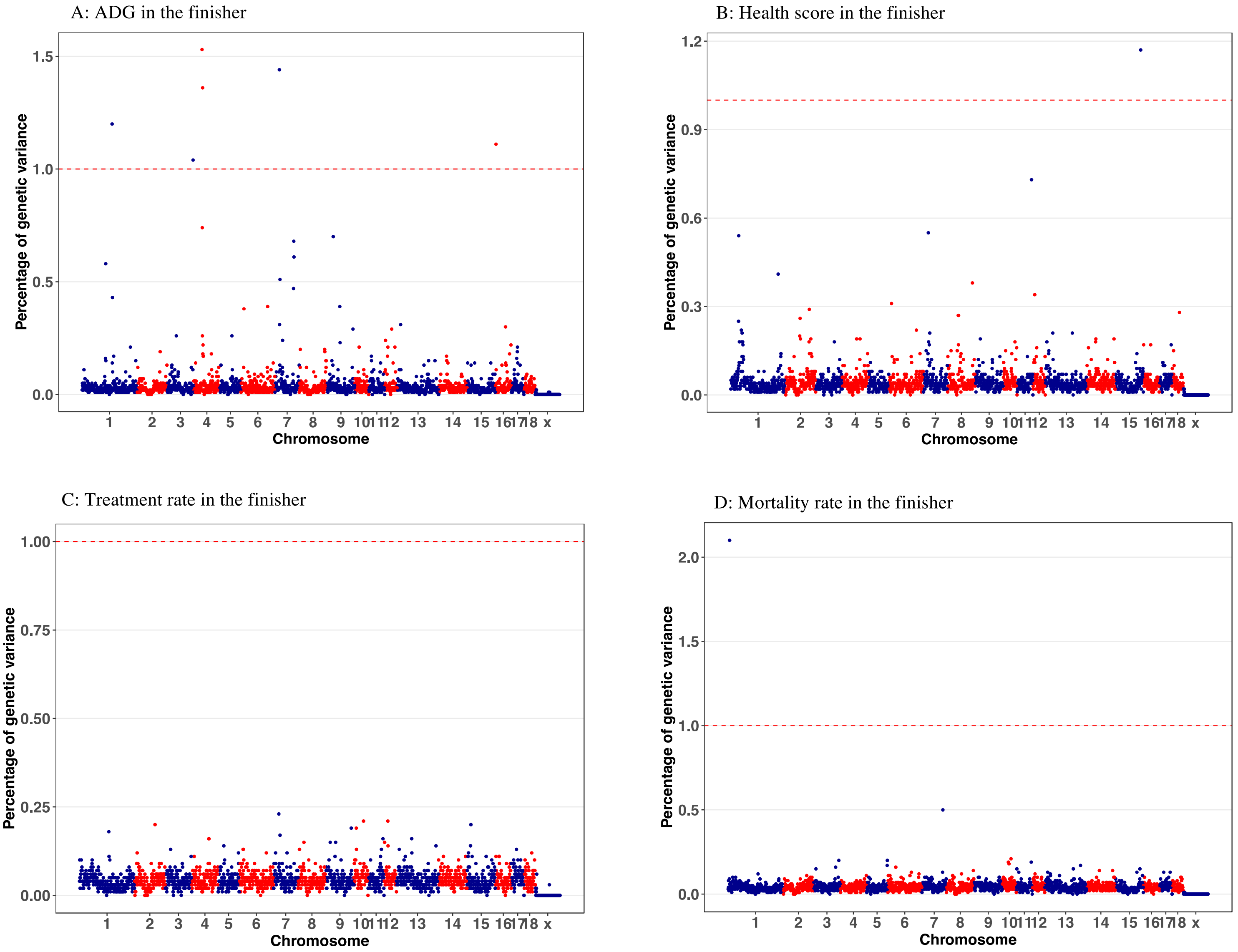

Figure S3. Percentage of genetic variance explained by each non-overlapping 1 Mb window across the genome for average daily gain (ADG), treatment rate, mortality rate, and health score in the finisher based on univariate GWAS

Each dot indicates 1-Mb genomic window and univariate GWAS was performed using BayesB with Pi = 0.999.

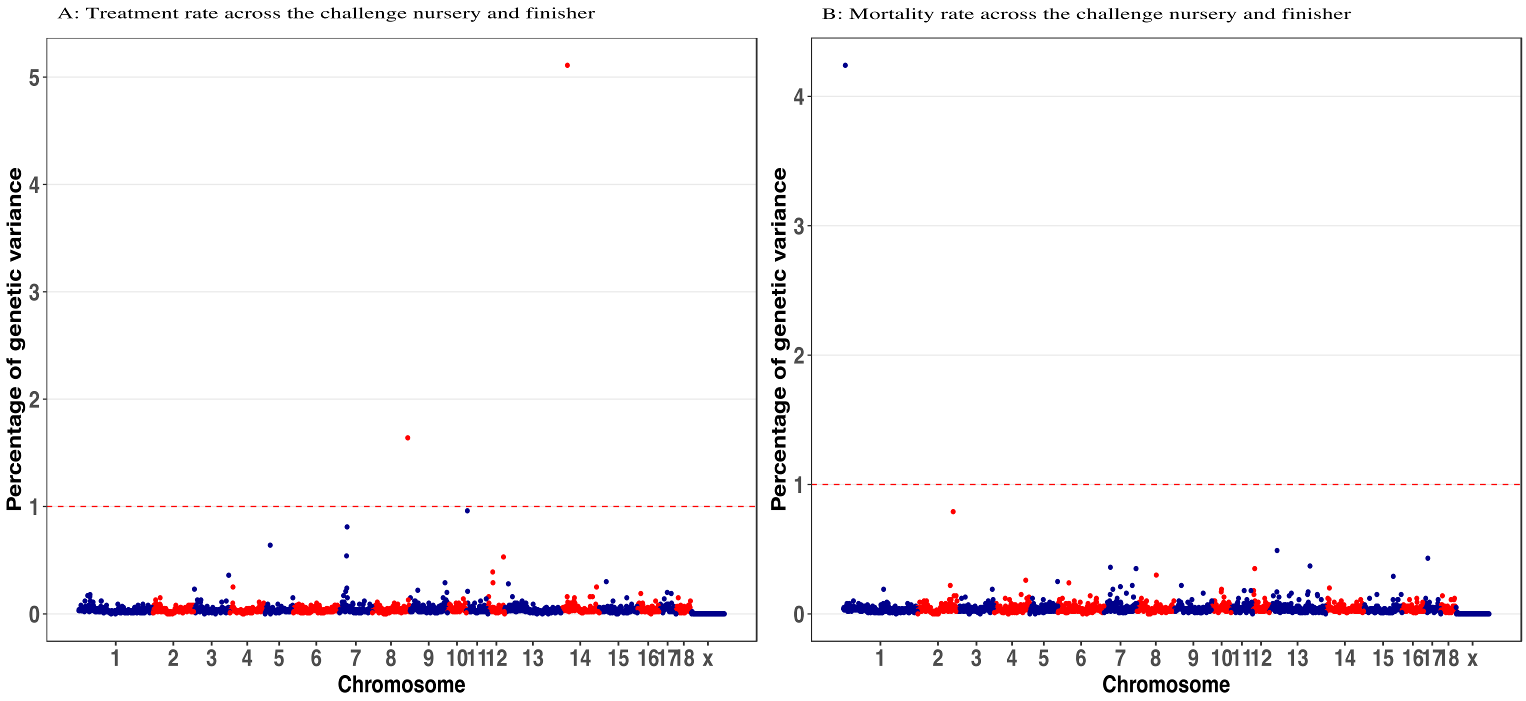

Figure S4. Percentage of genetic variance explained by each non-overlapping 1 Mb window across the genome for treatment rate and mortality rate across the challenge nursery and finisher based on univariate GWAS

Each dot indicates 1-Mb genomic window and univariate GWAS was performed using BayesB with Pi = 0.999.

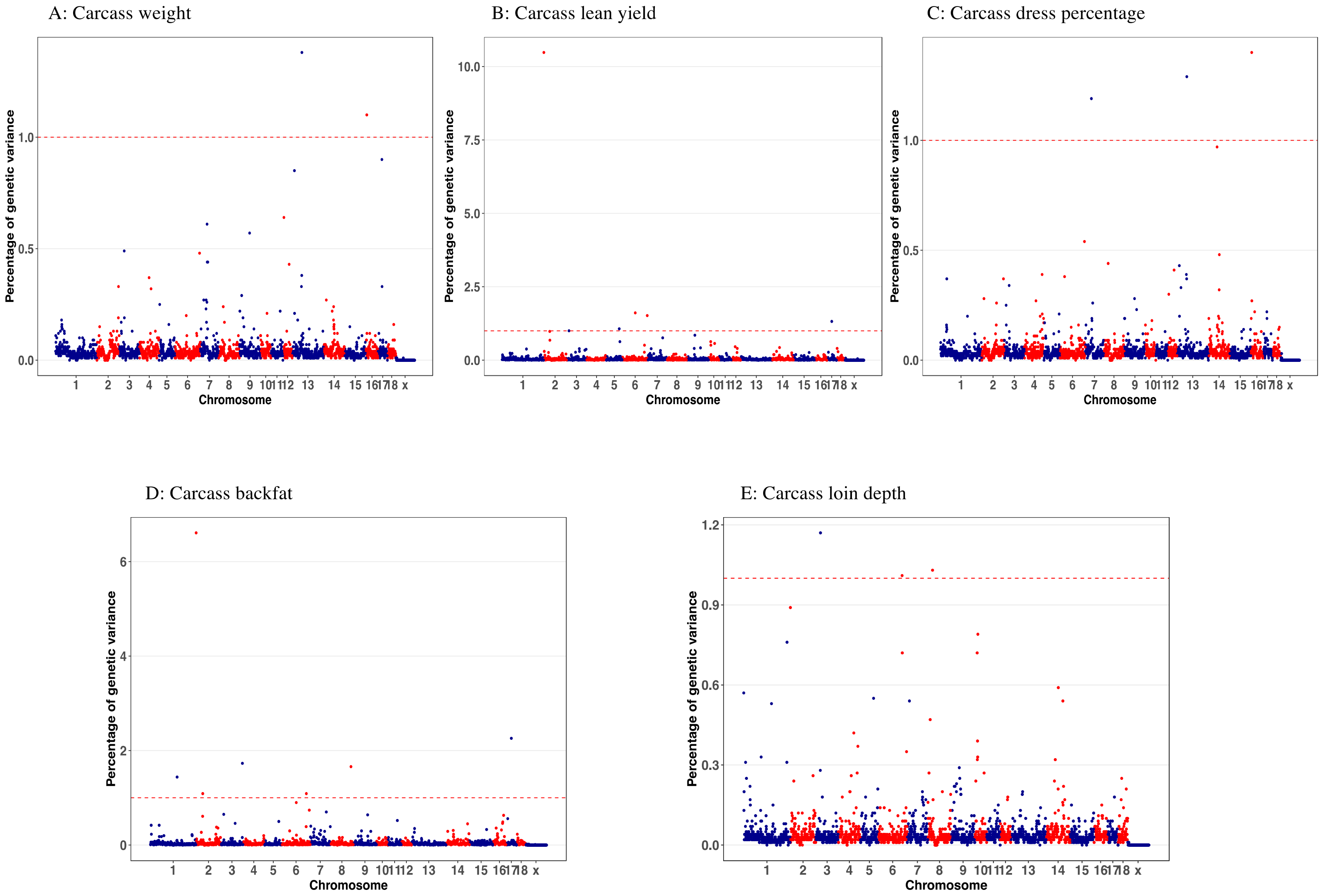

Figure S5. Percentage of genetic variance explained by each non-overlapping 1 Mb window across the genome for carcass traits based on univariate GWAS

Each dot indicates 1-Mb genomic window and univariate GWAS was performed using BayesB with Pi = 0.999.

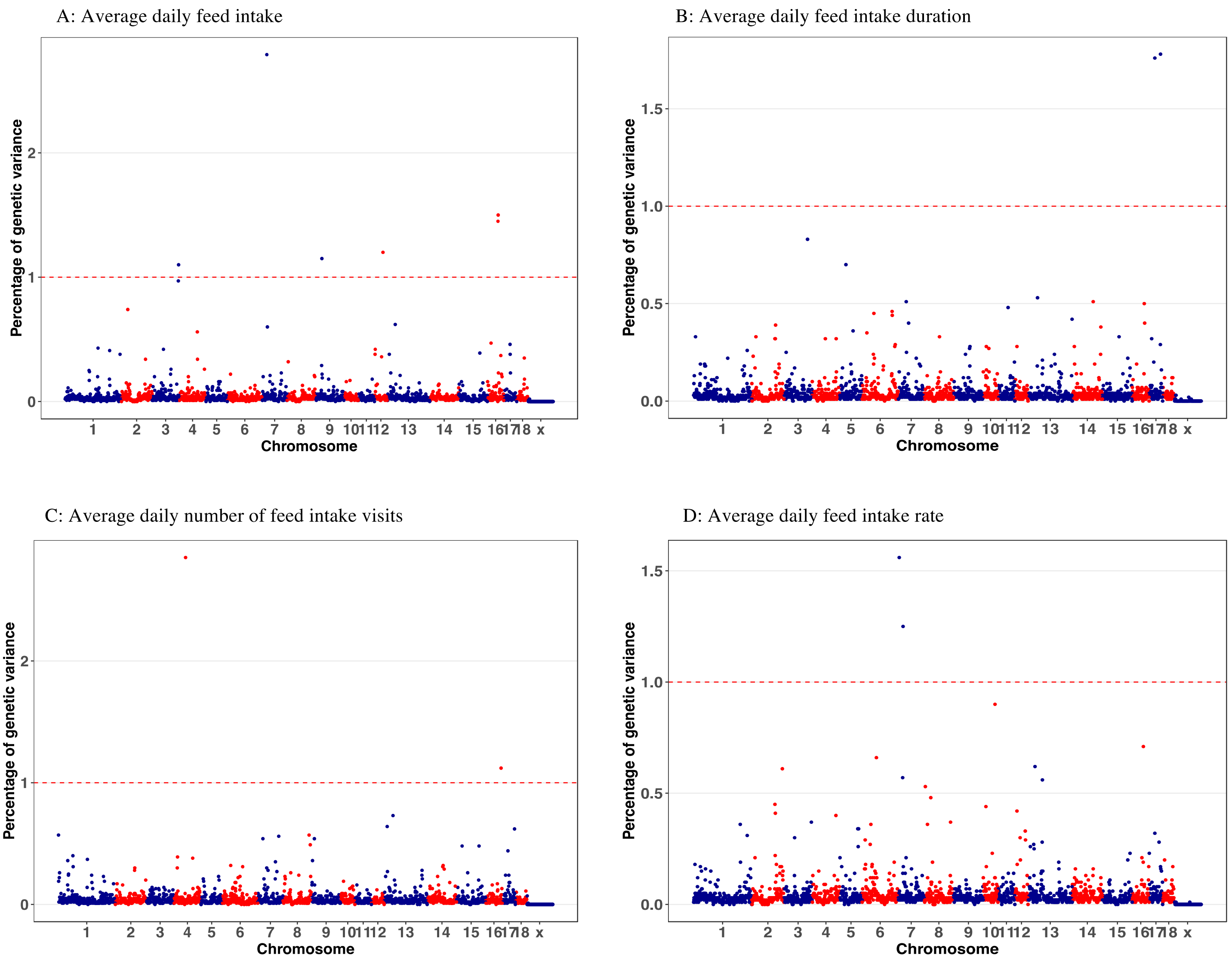

Figure S6. Percentage of genetic variance explained by each non-overlapping 1 Mb window across the genome for feed intake and behavior traits based on univariate GWAS

Each dot indicates 1-Mb genomic window and univariate GWAS was performed using BayesB with Pi = 0.999.

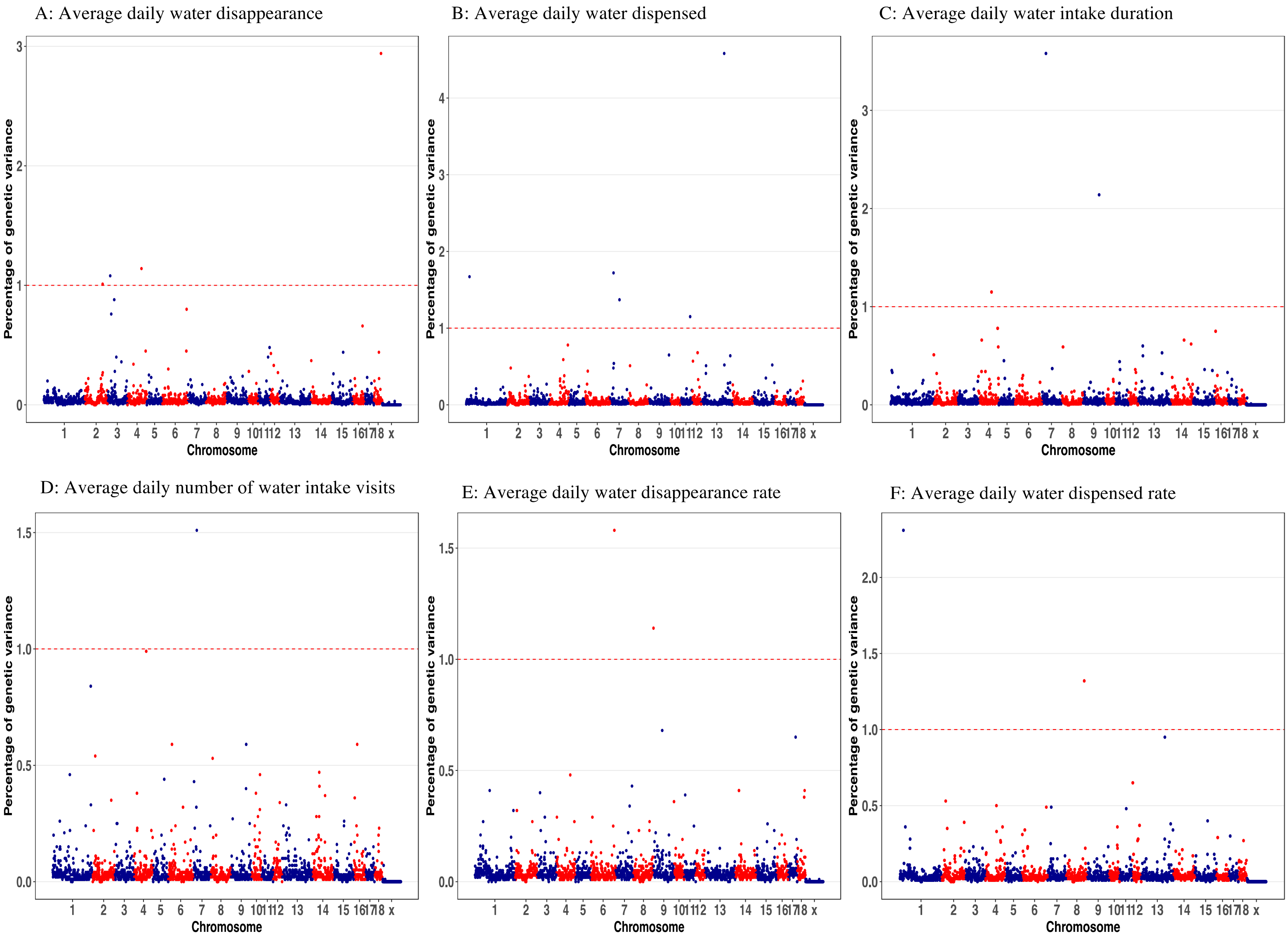

Figure S7. Percentage of genetic variance explained by each non-overlapping 1 Mb window across the genome for water intake and behavior traits based on univariate GWAS

Each dot indicates 1-Mb genomic window and univariate GWAS was performed using BayesB with Pi = 0.999.

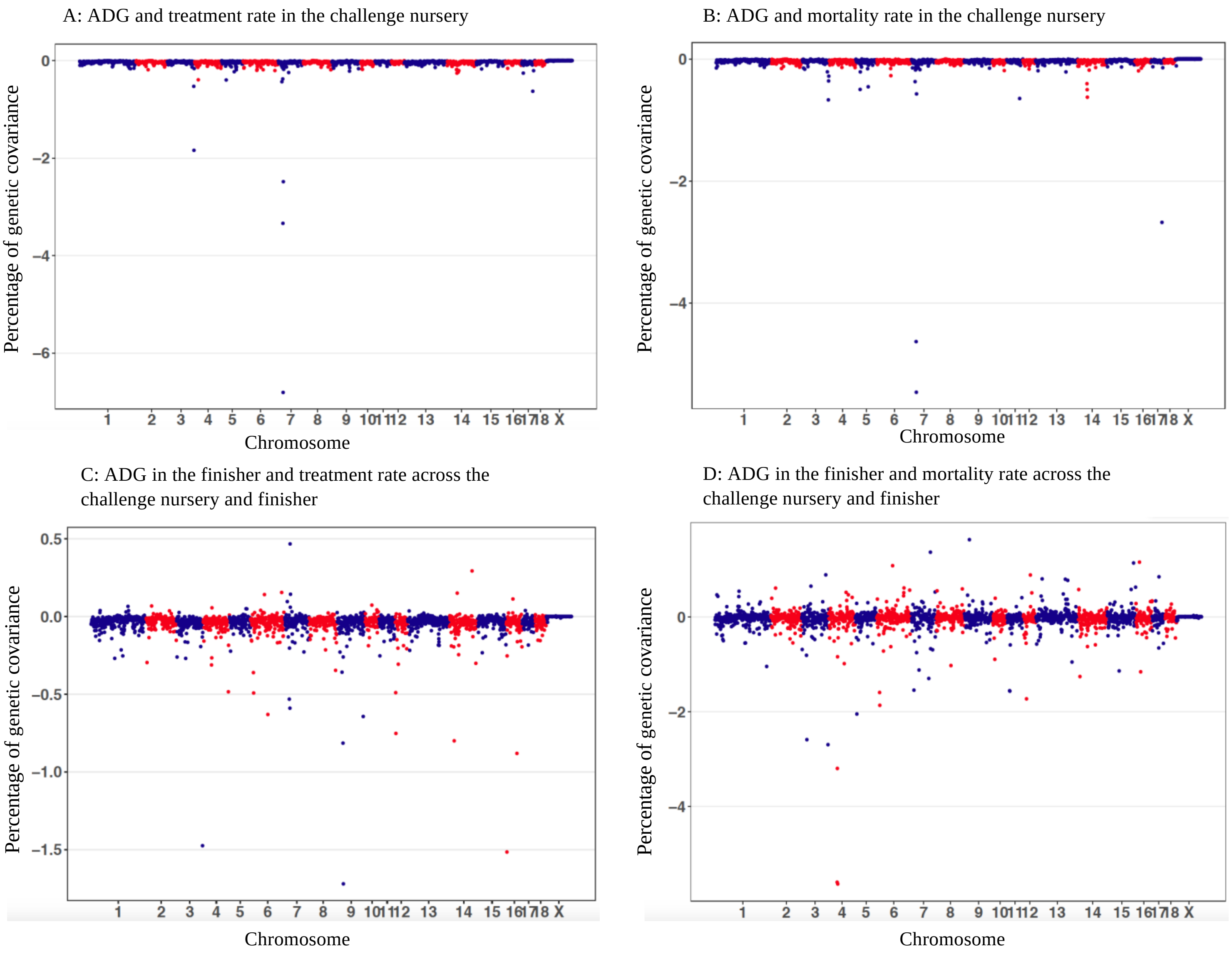

Figure S8. Percentage of genetic covariance explained by each non-overlapping 1 Mb window across the genome for average daily gain (ADG) with treatment rate and mortality rate based on bivariate GWAS Each dot indicates 1-Mb genomic window and bivariate GWAS was performed using BayesB.

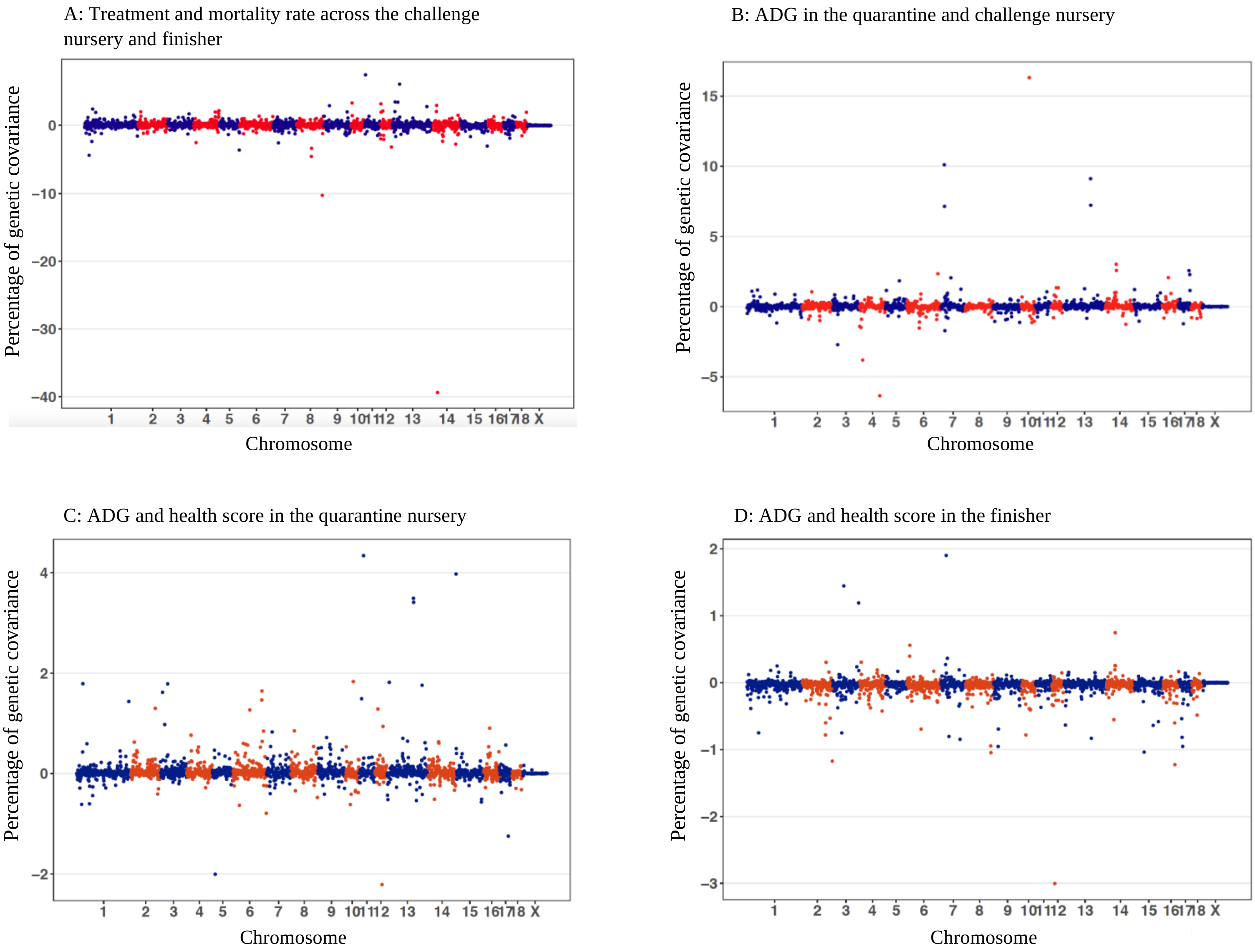

Figure S9. Percentage of genetic covariance explained by each non-overlapping 1 Mb window across the genome for treatment rate with mortality rate and average daily gain (ADG) in the quarantine nursery with ADG in the challenge nursery and ADG with health score in each phase based on bivariate GWAS Each dot indicates 1-Mb genomic window and bivariate GWAS was performed using BayesB.

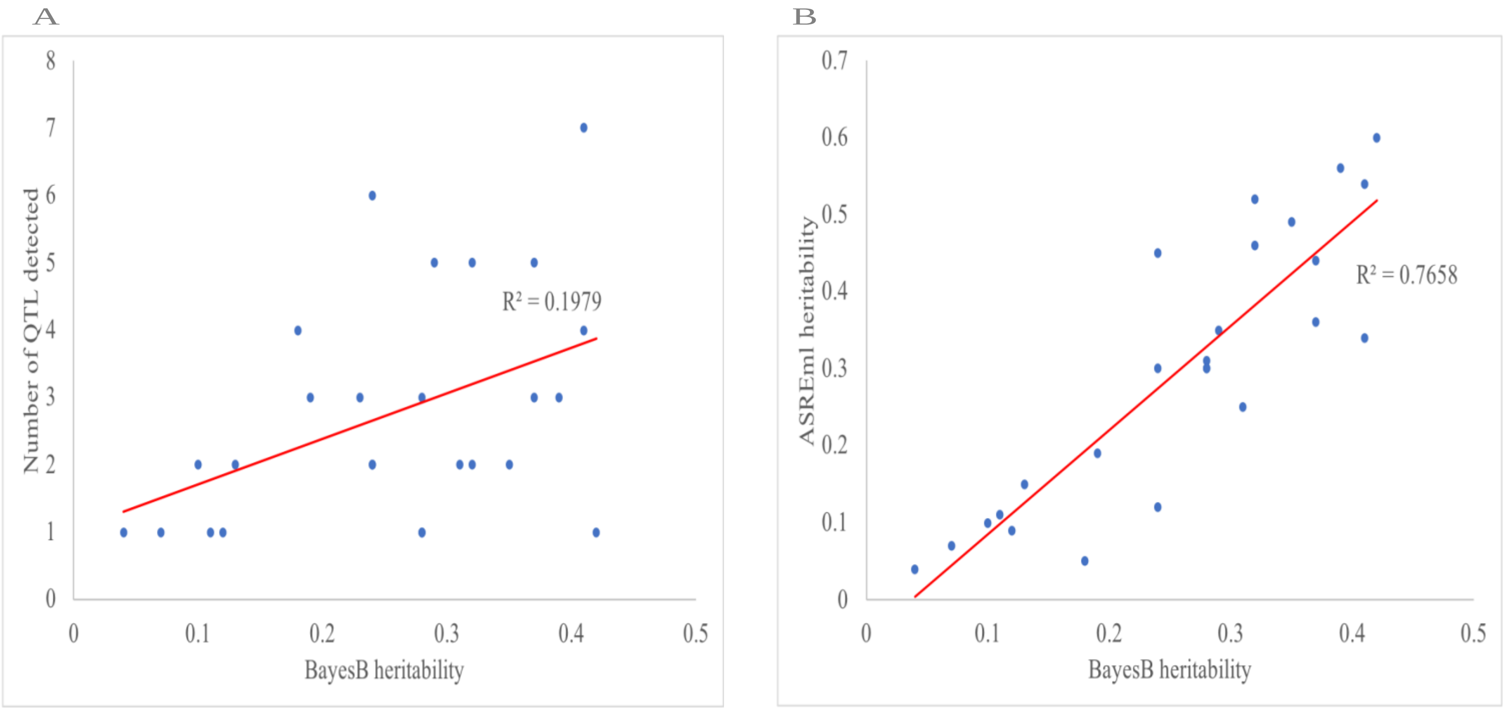

Figure S10. Relationship of BayesB heritability with number of quantitative trait loci (QTL) detected by univariate GWAS and with ASReml heritability

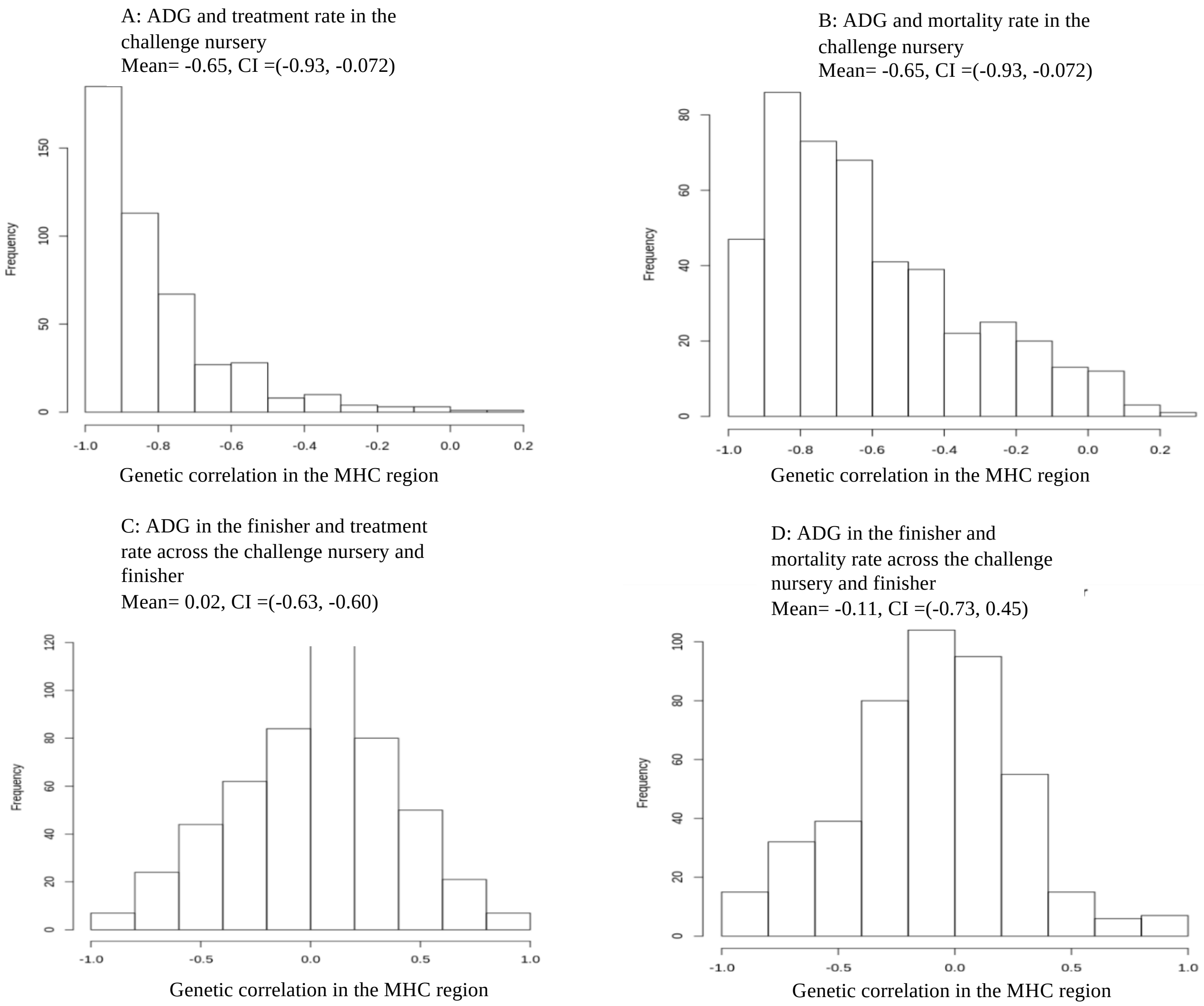

Figure S11. Posterior distribution of genetic correlation in the major histocompatibility complex (MHC) region for average daily gain (ADG) with treatment rate and mortality rate based on bivariate GWAS Bivariate GWAS was performed using BayesB model. CI: 95% credible interval; MHC: major histocompatibility complex.

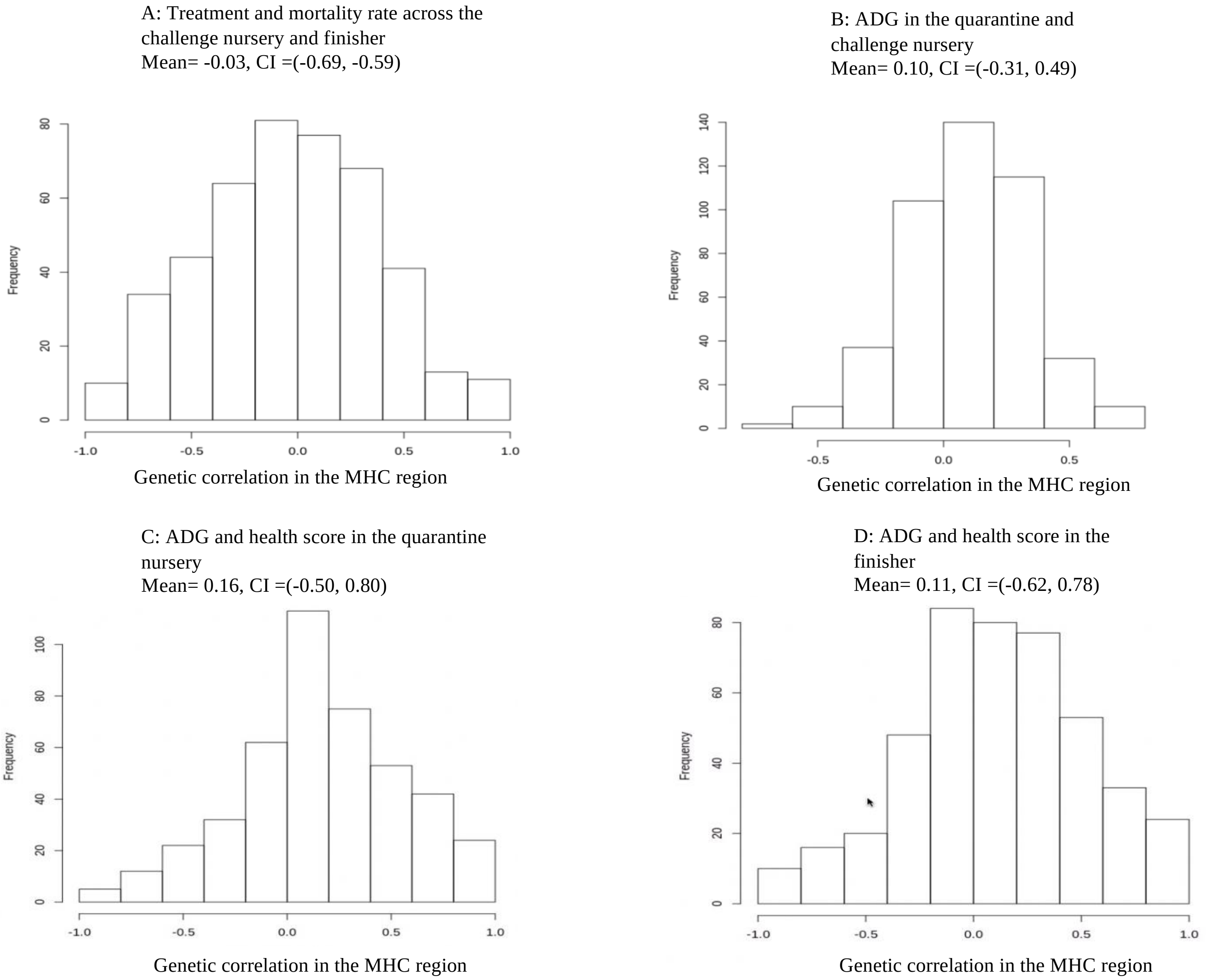

Figure S12. Posterior distribution of genetic correlation in the major histocompatibility complex (MHC) region for treatment rate with mortality rate and average daily gain (ADG) in the quarantine nursery with ADG in the challenge nursery and ADG with health score in each phase based on bivariate GWAS Bivariate GWAS was performed using BayesB model. CI: 95% credible interval; MHC: major histocompatibility complex.

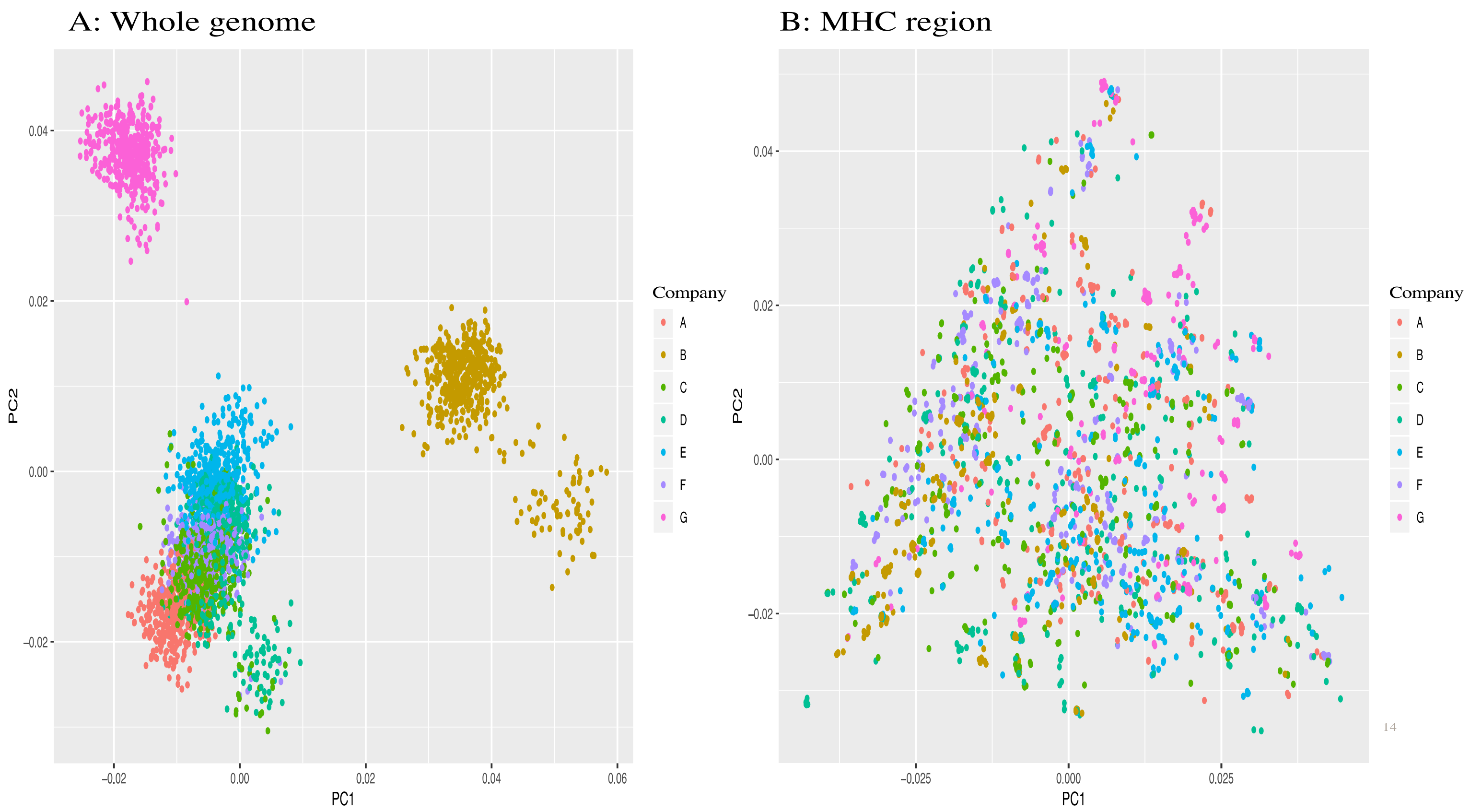

Figure S13. Principle component analysis (PCA) for genotypes in the whole genome and in the major histocompatibility complex (MHC).

PC: principle component; MHC: major histocompatibility complex.

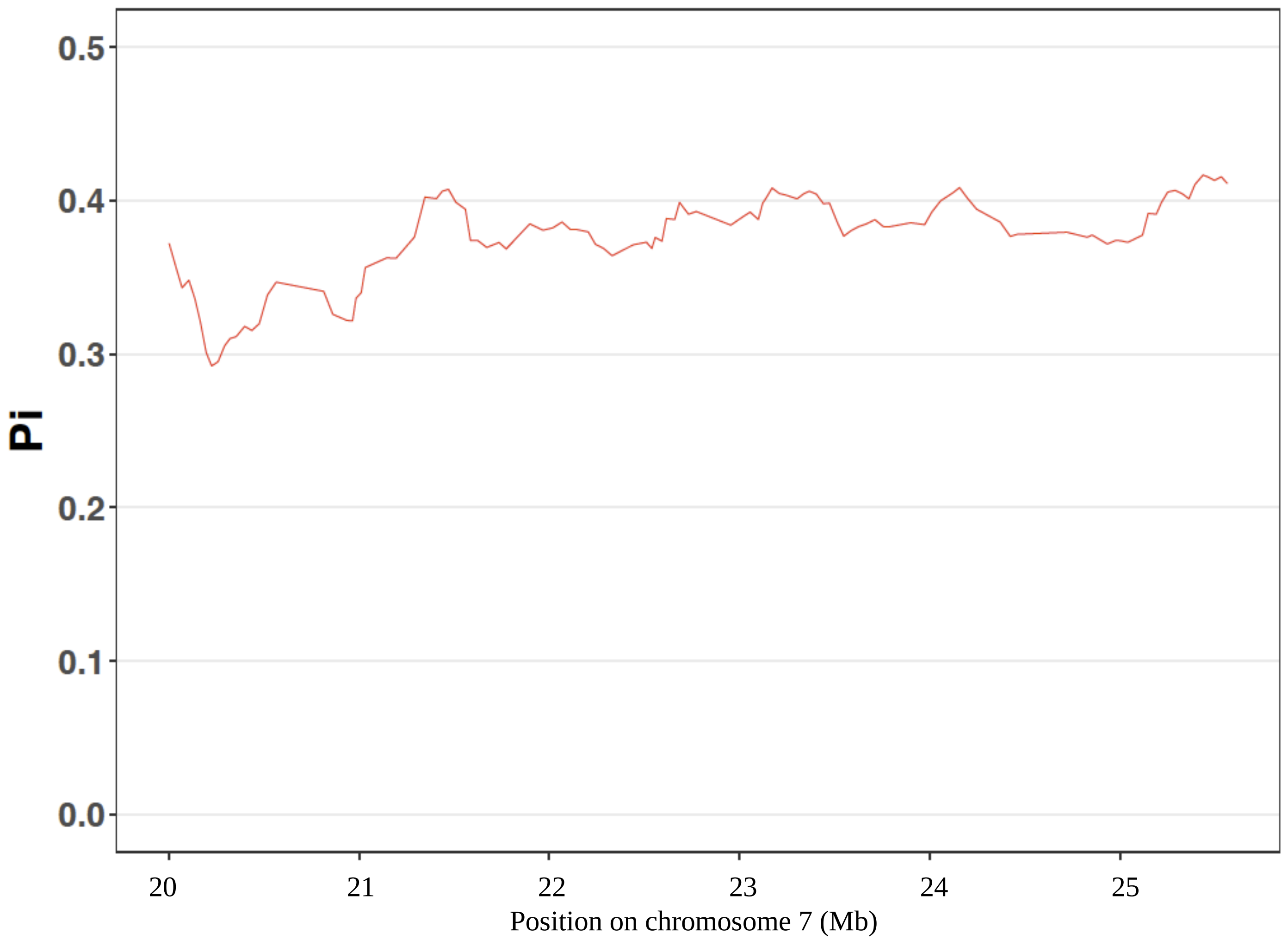

Figure S14. Single nucleotide polymorphism (SNP) diversity in the major histocompatibility complex (MHC) region

Pi: The average pairwise divergence among genotypes, representing the nucleotide diversity per bp.

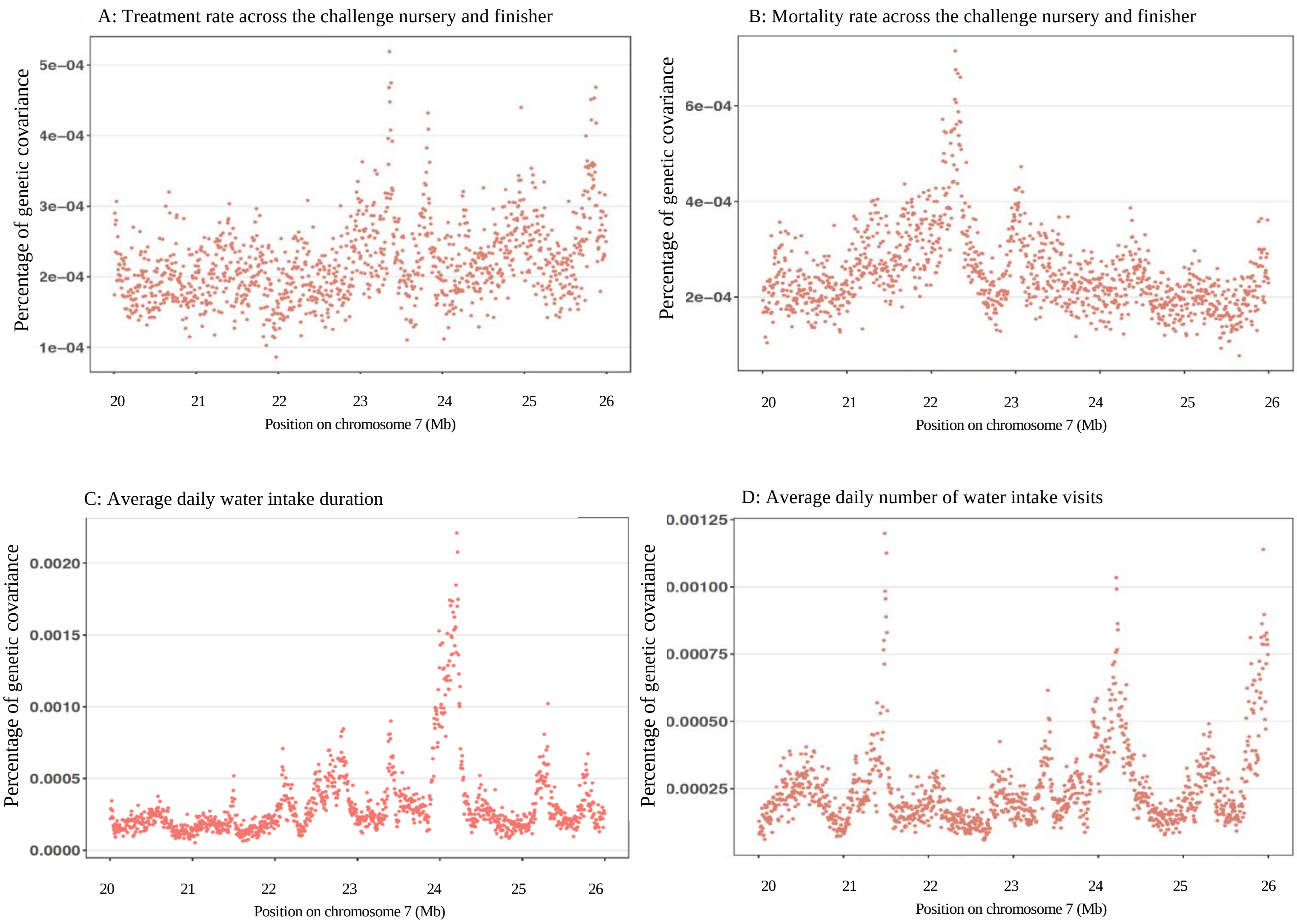

Figure S15. Quantitative trait loci (QTL) detected in the major histocompatibility complex region for treatment and mortality rate across the challenge nursery and finisher and water intake traits based on fine mapping using haplotype GWAS model

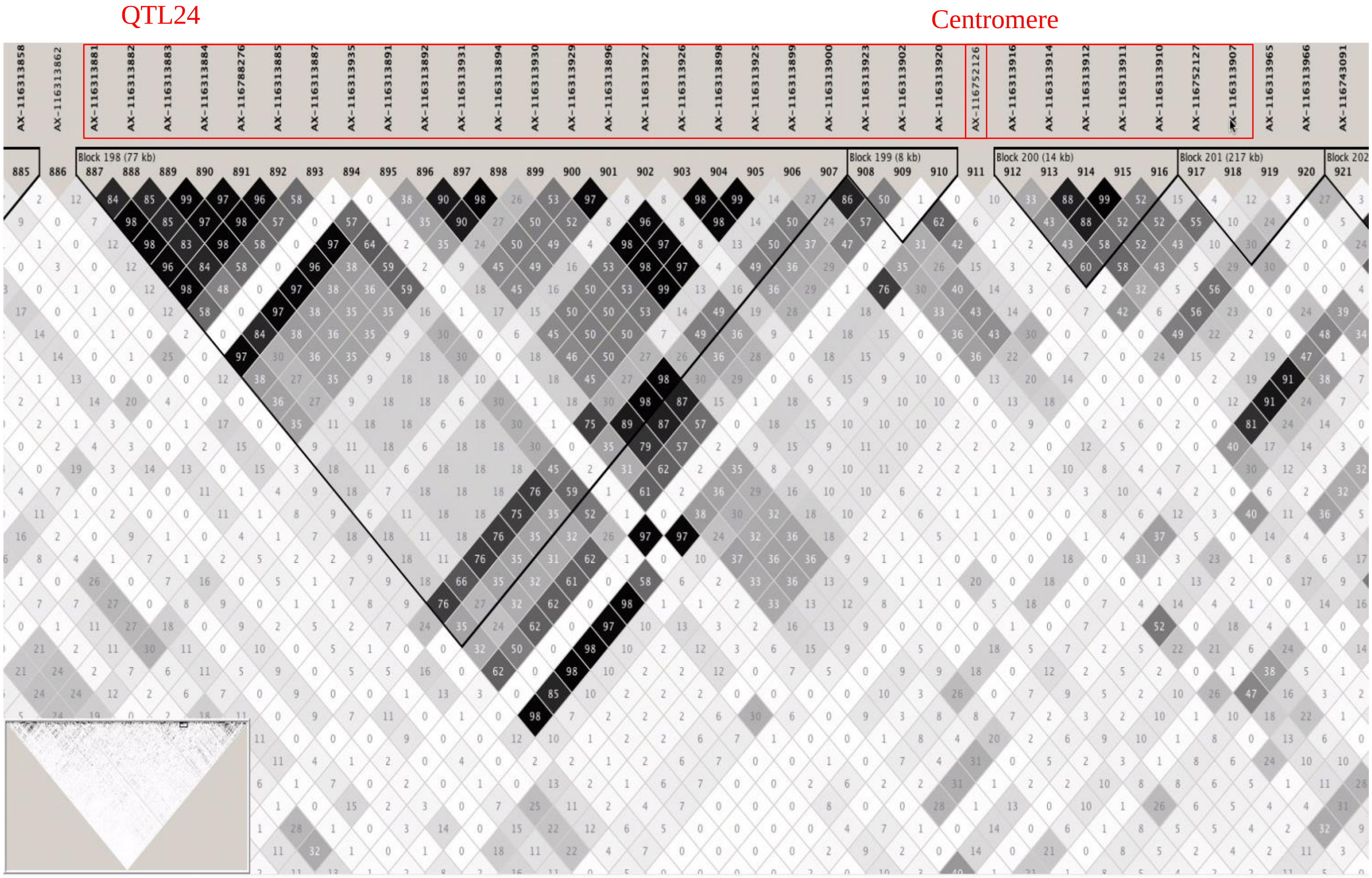

Figure S16. Linkage disequilibrium (r^2) plot for QTL24 identified for average daily gain (ADG) in the challenge nursery based on fine mapping using BayesC haplotype GWAS model with Pi = 0.999

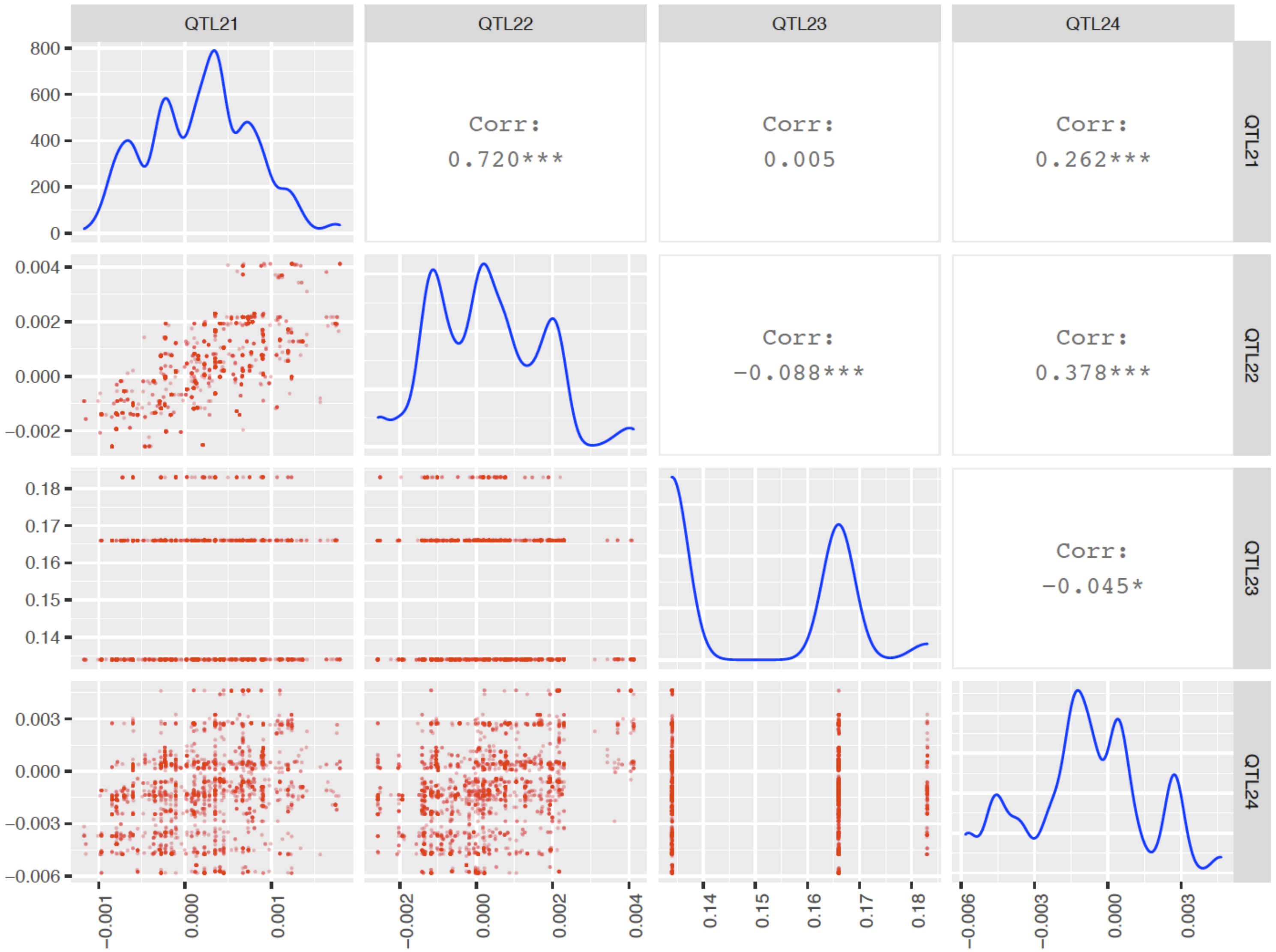

Figure S17. Estimated breeding values for each quantitative trait loci (QTL) in the major histocompatibility complex (MHC) region identified for average daily gain (ADG) in the challenge nursery based on fine mapping using haplotype GWAS model and genetic correlations among these QTL

Table S1. Variance components estimates for average daily gain (ADG) in the challenge nursery using ASReml, JWAS, and BayesIM

**Method**	$\boldsymbol{\sigma_g^2}$	$\boldsymbol{\sigma_e^2}$	$\boldsymbol{\sigma_s^2}$	$\boldsymbol{\sigma_p^2}$	$\boldsymbol{h^2}$	**%gVar for MHC**
ASReml (GBLUP)	0.003	0.012	0.0009	0.001	0.19	NA
JWAS (Pi=0.999)	0.003	0.012	0.0008	0.001	0.22	5
BayesIM (8 haplotype clusters, Pi=0.99, 20 QTL/Mb)	0.004	0.012	0.0009	0.001	0.23	1.78
BayesIM (8 haplotype clusters, Pi= 0.999, 200 QTL/Mb)	0.004	0.011	0.0007	0.001	0.27	1.58
BayesIM (16 haplotype clusters, Pi=0.99, 20 QTL/Mb)	0.004	0.011	0.0007	0.001	0.27	1.58

σ_g^2: genetic variance; σ_g^2: residual variance; σ_g^2: litter effect variance; σ_g^2: pen effect variance; h^2: heritability; %gVar: percentage of genetic variance; GBLUP: genomic best linear unbiased prediction; QTL: quantitative trait loci; MHC: major histocompatibility complex; Pi: prior probability of QTL has zero effect.
